# Supplementary material for: Genome-wide identification, characterization and gene expression of BES1 transcription factor family in grapevine (Vitis vinifera L.)
Source: Sci Rep. 2023 Jan 5;13:240. doi: 10.1038/s41598-022-24407-y (PMC9816167; doi:10.1038/s41598-022-24407-y)
Supplement: Supplementary file 3 — Supplementary Information. [file 41598_2022_24407_MOESM3_ESM.zip › Chromosome collinearity analysis/MCsanX.out.html.zip!/MCsanX.out.html/2.html]

|  |  |  |  |  |  |  |  |  |  |  |  |  |  |
| --- | --- | --- | --- | --- | --- | --- | --- | --- | --- | --- | --- | --- | --- |
| Duplication depth | Reference chromosome | Collinear blocks | | | | | | | | | | | |
| 1 | transcript:Vitvi02g00001\_t001 |  | transcript:Vitvi15g01059\_t001 |  |  |  |  |  |  |  |  |  |  |
| 1 | transcript:Vitvi02g00002\_t001 |  | \| \| |  |  |  |  |  |  |  |  |  |  |
| 1 | transcript:Vitvi02g04000\_t001 |  | \| \| |  |  |  |  |  |  |  |  |  |  |
| 1 | transcript:Vitvi02g04001\_t001 |  | \| \| |  |  |  |  |  |  |  |  |  |  |
| 1 | transcript:Vitvi02g00006\_t001 |  | \| \| |  |  |  |  |  |  |  |  |  |  |
| 1 | transcript:Vitvi02g04002\_t001 |  | \| \| |  |  |  |  |  |  |  |  |  |  |
| 1 | transcript:Vitvi02g00007\_t001 |  | \| \| |  |  |  |  |  |  |  |  |  |  |
| 1 | transcript:Vitvi02g00008\_t001 |  | \| \| |  |  |  |  |  |  |  |  |  |  |
| 1 | transcript:Vitvi02g00009\_t001 |  | \| \| |  |  |  |  |  |  |  |  |  |  |
| 1 | transcript:Vitvi02g00011\_t001 |  | \| \| |  |  |  |  |  |  |  |  |  |  |
| 1 | transcript:Vitvi02g04003\_t002 |  | transcript:Vitvi15g01066\_t001 |  |  |  |  |  |  |  |  |  |  |
| 1 | transcript:Vitvi02g00013\_t001 |  | transcript:Vitvi15g01067\_t001 |  |  |  |  |  |  |  |  |  |  |
| 1 | transcript:Vitvi02g00014\_t001 |  | \| \| |  |  |  |  |  |  |  |  |  |  |
| 2 | transcript:Vitvi02g01312\_t001 |  | \| \| |  | transcript:Vitvi16g01089\_t001 |  |  |  |  |  |  |  |  |
| 2 | transcript:Vitvi02g04004\_t001 |  | \| \| |  | \| \| |  |  |  |  |  |  |  |  |
| 2 | transcript:Vitvi02g00015\_t001 |  | \| \| |  | \| \| |  |  |  |  |  |  |  |  |
| 2 | transcript:Vitvi02g04005\_t001 |  | \| \| |  | \| \| |  |  |  |  |  |  |  |  |
| 2 | transcript:Vitvi02g00016\_t001 |  | \| \| |  | \| \| |  |  |  |  |  |  |  |  |
| 2 | transcript:Vitvi02g00017\_t001 |  | \| \| |  | \| \| |  |  |  |  |  |  |  |  |
| 2 | transcript:Vitvi02g00018\_t001 |  | \| \| |  | \| \| |  |  |  |  |  |  |  |  |
| 2 | transcript:Vitvi02g04006\_t001 |  | \| \| |  | \| \| |  |  |  |  |  |  |  |  |
| 2 | transcript:Vitvi02g01313\_t001 |  | \| \| |  | \| \| |  |  |  |  |  |  |  |  |
| 2 | transcript:Vitvi02g00020\_t001 |  | \| \| |  | transcript:Vitvi16g01099\_t001 |  |  |  |  |  |  |  |  |
| 2 | transcript:Vitvi02g00021\_t001 |  | \| \| |  | \| \| |  |  |  |  |  |  |  |  |
| 2 | transcript:Vitvi02g00022\_t001 |  | transcript:Vitvi15g04607\_t001 |  | transcript:Vitvi16g01920\_t001 |  |  |  |  |  |  |  |  |
| 2 | transcript:Vitvi02g00023\_t001 |  | transcript:Vitvi15g01077\_t001 |  | \| \| |  |  |  |  |  |  |  |  |
| 2 | transcript:Vitvi02g00025\_t001 |  | \| \| |  | transcript:Vitvi16g01103\_t001 |  |  |  |  |  |  |  |  |
| 2 | transcript:Vitvi02g01314\_t001 |  | \| \| |  | \| \| |  |  |  |  |  |  |  |  |
| 2 | transcript:Vitvi02g01315\_t001 |  | \| \| |  | transcript:Vitvi16g01104\_t001 |  |  |  |  |  |  |  |  |
| 2 | transcript:Vitvi02g00028\_t001 |  | \| \| |  | \| \| |  |  |  |  |  |  |  |  |
| 2 | transcript:Vitvi02g00029\_t001 |  | \| \| |  | \| \| |  |  |  |  |  |  |  |  |
| 2 | transcript:Vitvi02g00030\_t001 |  | \| \| |  | \| \| |  |  |  |  |  |  |  |  |
| 2 | transcript:Vitvi02g00031\_t001 |  | \| \| |  | transcript:Vitvi16g04379\_t001 |  |  |  |  |  |  |  |  |
| 1 | transcript:Vitvi02g00032\_t001 |  | \| \| |  |  |  |  |  |  |  |  |  |  |
| 1 | transcript:Vitvi02g00034\_t001 |  | \| \| |  |  |  |  |  |  |  |  |  |  |
| 1 | transcript:Vitvi02g00035\_t001 |  | \| \| |  |  |  |  |  |  |  |  |  |  |
| 1 | transcript:Vitvi02g00037\_t002 |  | \| \| |  |  |  |  |  |  |  |  |  |  |
| 1 | transcript:Vitvi02g00038\_t001 |  | \| \| |  |  |  |  |  |  |  |  |  |  |
| 2 | transcript:Vitvi02g00039\_t001 |  | \| \| |  | transcript:Vitvi16g01132\_t001 |  |  |  |  |  |  |  |  |
| 2 | transcript:Vitvi02g00040\_t001 |  | \| \| |  | \| \| |  |  |  |  |  |  |  |  |
| 2 | transcript:Vitvi02g00041\_t001 |  | \| \| |  | \| \| |  |  |  |  |  |  |  |  |
| 2 | transcript:Vitvi02g00042\_t001 |  | \| \| |  | transcript:Vitvi16g01136\_t003 |  |  |  |  |  |  |  |  |
| 2 | transcript:Vitvi02g00043\_t001 |  | transcript:Vitvi15g01648\_t001 |  | \| \| |  |  |  |  |  |  |  |  |
| 1 | transcript:Vitvi02g00044\_t001 |  |  |  | \| \| |  |  |  |  |  |  |  |  |
| 1 | transcript:Vitvi02g00045\_t001 |  |  |  | \| \| |  |  |  |  |  |  |  |  |
| 1 | transcript:Vitvi02g00046\_t001 |  |  |  | \| \| |  |  |  |  |  |  |  |  |
| 1 | transcript:Vitvi02g00048\_t001 |  |  |  | \| \| |  |  |  |  |  |  |  |  |
| 1 | transcript:Vitvi02g01317\_t001 |  |  |  | transcript:Vitvi16g01146\_t001 |  |  |  |  |  |  |  |  |
| 1 | transcript:Vitvi02g01318\_t001 |  |  |  | transcript:Vitvi16g01952\_t001 |  |  |  |  |  |  |  |  |
| 1 | transcript:Vitvi02g01319\_t001 |  |  |  | \| \| |  |  |  |  |  |  |  |  |
| 1 | transcript:Vitvi02g04007\_t001 |  |  |  | \| \| |  |  |  |  |  |  |  |  |
| 1 | transcript:Vitvi02g00051\_t001 |  |  |  | transcript:Vitvi16g04397\_t001 |  |  |  |  |  |  |  |  |
| 1 | transcript:Vitvi02g00052\_t001 |  |  |  | \| \| |  |  |  |  |  |  |  |  |
| 1 | transcript:Vitvi02g00053\_t001 |  |  |  | \| \| |  |  |  |  |  |  |  |  |
| 1 | transcript:Vitvi02g00054\_t001 |  |  |  | \| \| |  |  |  |  |  |  |  |  |
| 1 | transcript:Vitvi02g00055\_t001 |  |  |  | \| \| |  |  |  |  |  |  |  |  |
| 1 | transcript:Vitvi02g00056\_t001 |  |  |  | \| \| |  |  |  |  |  |  |  |  |
| 1 | transcript:Vitvi02g01321\_t001 |  |  |  | \| \| |  |  |  |  |  |  |  |  |
| 2 | transcript:Vitvi02g00059\_t001 |  | transcript:Vitvi15g01640\_t001.1.6037826c |  | transcript:Vitvi16g01161\_t001 |  |  |  |  |  |  |  |  |
| 2 | transcript:Vitvi02g00060\_t001 |  | \| \| |  | transcript:Vitvi16g01957\_t001 |  |  |  |  |  |  |  |  |
| 2 | transcript:Vitvi02g00062\_t001 |  | \| \| |  | transcript:Vitvi16g01166\_t001 |  |  |  |  |  |  |  |  |
| 1 | transcript:Vitvi02g00063\_t001 |  | \| \| |  |  |  |  |  |  |  |  |  |  |
| 1 | transcript:Vitvi02g00065\_t002 |  | \| \| |  |  |  |  |  |  |  |  |  |  |
| 1 | transcript:Vitvi02g00066\_t001 |  | \| \| |  |  |  |  |  |  |  |  |  |  |
| 1 | transcript:Vitvi02g00067\_t001 |  | \| \| |  |  |  |  |  |  |  |  |  |  |
| 1 | transcript:Vitvi02g04008\_t001 |  | \| \| |  |  |  |  |  |  |  |  |  |  |
| 1 | transcript:Vitvi02g00068\_t001 |  | \| \| |  |  |  |  |  |  |  |  |  |  |
| 1 | transcript:Vitvi02g00069\_t001 |  | \| \| |  |  |  |  |  |  |  |  |  |  |
| 1 | transcript:Vitvi02g00070\_t001 |  | \| \| |  |  |  |  |  |  |  |  |  |  |
| 1 | transcript:Vitvi02g01323\_t001 |  | \| \| |  |  |  |  |  |  |  |  |  |  |
| 1 | transcript:Vitvi02g01324\_t001 |  | \| \| |  |  |  |  |  |  |  |  |  |  |
| 1 | transcript:Vitvi02g01325\_t001 |  | \| \| |  |  |  |  |  |  |  |  |  |  |
| 1 | transcript:Vitvi02g00071\_t001 |  | \| \| |  |  |  |  |  |  |  |  |  |  |
| 1 | transcript:Vitvi02g04009\_t001 |  | \| \| |  |  |  |  |  |  |  |  |  |  |
| 1 | transcript:Vitvi02g04010\_t001 |  | \| \| |  |  |  |  |  |  |  |  |  |  |
| 1 | transcript:Vitvi02g01327\_t001 |  | \| \| |  |  |  |  |  |  |  |  |  |  |
| 1 | transcript:Vitvi02g01328\_t001 |  | \| \| |  |  |  |  |  |  |  |  |  |  |
| 1 | transcript:Vitvi02g00073\_t001 |  | \| \| |  |  |  |  |  |  |  |  |  |  |
| 1 | transcript:Vitvi02g00075\_t001 |  | transcript:Vitvi15g01033\_t003 |  |  |  |  |  |  |  |  |  |  |
| 1 | transcript:Vitvi02g04011\_t001 |  | \| \| |  |  |  |  |  |  |  |  |  |  |
| 1 | transcript:Vitvi02g01329\_t001 |  | transcript:Vitvi15g01032\_t001 |  |  |  |  |  |  |  |  |  |  |
| 1 | transcript:Vitvi02g00076\_t001 |  | \| \| |  |  |  |  |  |  |  |  |  |  |
| 1 | transcript:Vitvi02g00077\_t001 |  | \| \| |  |  |  |  |  |  |  |  |  |  |
| 1 | transcript:Vitvi02g00078\_t001 |  | transcript:Vitvi15g01031\_t001 |  |  |  |  |  |  |  |  |  |  |
| 1 | transcript:Vitvi02g00079\_t001 |  | transcript:Vitvi15g01029\_t001 |  |  |  |  |  |  |  |  |  |  |
| 1 | transcript:Vitvi02g00080\_t001 |  | \| \| |  |  |  |  |  |  |  |  |  |  |
| 1 | transcript:Vitvi02g00081\_t001 |  | \| \| |  |  |  |  |  |  |  |  |  |  |
| 1 | transcript:Vitvi02g01331\_t001 |  | \| \| |  |  |  |  |  |  |  |  |  |  |
| 2 | transcript:Vitvi02g00082\_t002 |  | \| \| |  | transcript:Vitvi16g01210\_t005 |  |  |  |  |  |  |  |  |
| 2 | transcript:Vitvi02g01333\_t001 |  | \| \| |  | \| \| |  |  |  |  |  |  |  |  |
| 2 | transcript:Vitvi02g00083\_t001 |  | \| \| |  | \| \| |  |  |  |  |  |  |  |  |
| 2 | transcript:Vitvi02g00085\_t001 |  | \| \| |  | \| \| |  |  |  |  |  |  |  |  |
| 2 | transcript:Vitvi02g00086\_t001 |  | \| \| |  | \| \| |  |  |  |  |  |  |  |  |
| 2 | transcript:Vitvi02g00087\_t001 |  | \| \| |  | \| \| |  |  |  |  |  |  |  |  |
| 2 | transcript:Vitvi02g00088\_t001 |  | \| \| |  | transcript:Vitvi16g01207\_t002 |  |  |  |  |  |  |  |  |
| 2 | transcript:Vitvi02g04012\_t001 |  | \| \| |  | \| \| |  |  |  |  |  |  |  |  |
| 2 | transcript:Vitvi02g00089\_t001 |  | transcript:Vitvi15g01027\_t001 |  | \| \| |  |  |  |  |  |  |  |  |
| 2 | transcript:Vitvi02g00090\_t001 |  | transcript:Vitvi15g01026\_t001 |  | \| \| |  |  |  |  |  |  |  |  |
| 2 | transcript:Vitvi02g00091\_t003 |  | \| \| |  | transcript:Vitvi16g01204\_t001 |  |  |  |  |  |  |  |  |
| 2 | transcript:Vitvi02g04013\_t001 |  | transcript:Vitvi15g04595\_t001 |  | transcript:Vitvi16g04430\_t001 |  |  |  |  |  |  |  |  |
| 2 | transcript:Vitvi02g00093\_t001 |  | transcript:Vitvi15g01021\_t001 |  | transcript:Vitvi16g01201\_t001 |  |  |  |  |  |  |  |  |
| 2 | transcript:Vitvi02g01334\_t001 |  | \| \| |  | transcript:Vitvi16g01198\_t001 |  |  |  |  |  |  |  |  |
| 2 | transcript:Vitvi02g00095\_t001 |  | \| \| |  | \| \| |  |  |  |  |  |  |  |  |
| 2 | transcript:Vitvi02g01335\_t001 |  | transcript:Vitvi15g01019\_t001 |  | transcript:Vitvi16g01191\_t001 |  |  |  |  |  |  |  |  |
| 2 | transcript:Vitvi02g04014\_t001 |  | \| \| |  | \| \| |  |  |  |  |  |  |  |  |
| 2 | transcript:Vitvi02g00096\_t001 |  | transcript:Vitvi15g01629\_t001 |  | transcript:Vitvi16g01982\_t001 |  |  |  |  |  |  |  |  |
| 1 | transcript:Vitvi02g00097\_t001 |  | transcript:Vitvi15g01628\_t001 |  |  |  |  |  |  |  |  |  |  |
| 1 | transcript:Vitvi02g00098\_t001 |  | transcript:Vitvi15g01016\_t002 |  |  |  |  |  |  |  |  |  |  |
| 1 | transcript:Vitvi02g01336\_t001 |  | \| \| |  |  |  |  |  |  |  |  |  |  |
| 1 | transcript:Vitvi02g00099\_t001 |  | \| \| |  |  |  |  |  |  |  |  |  |  |
| 1 | transcript:Vitvi02g00100\_t001 |  | \| \| |  |  |  |  |  |  |  |  |  |  |
| 1 | transcript:Vitvi02g04015\_t001 |  | \| \| |  |  |  |  |  |  |  |  |  |  |
| 1 | transcript:Vitvi02g00101\_t003 |  | \| \| |  |  |  |  |  |  |  |  |  |  |
| 1 | transcript:Vitvi02g00103\_t001 |  | transcript:Vitvi15g01015\_t001 |  |  |  |  |  |  |  |  |  |  |
| 1 | transcript:Vitvi02g04016\_t001 |  | transcript:Vitvi15g01626\_t001 |  |  |  |  |  |  |  |  |  |  |
| 1 | transcript:Vitvi02g00105\_t001 |  | transcript:Vitvi15g01013\_t001 |  |  |  |  |  |  |  |  |  |  |
| 1 | transcript:Vitvi02g00106\_t001 |  | transcript:Vitvi15g01011\_t001 |  |  |  |  |  |  |  |  |  |  |
| 1 | transcript:Vitvi02g00108\_t001 |  | \| \| |  |  |  |  |  |  |  |  |  |  |
| 1 | transcript:Vitvi02g00109\_t001 |  | \| \| |  |  |  |  |  |  |  |  |  |  |
| 1 | transcript:Vitvi02g01337\_t001 |  | \| \| |  |  |  |  |  |  |  |  |  |  |
| 1 | transcript:Vitvi02g00110\_t001 |  | transcript:Vitvi15g01623\_t001 |  |  |  |  |  |  |  |  |  |  |
| 1 | transcript:Vitvi02g00111\_t001 |  | \| \| |  |  |  |  |  |  |  |  |  |  |
| 1 | transcript:Vitvi02g00112\_t001 |  | transcript:Vitvi15g01005\_t001 |  |  |  |  |  |  |  |  |  |  |
| 1 | transcript:Vitvi02g00113\_t001 |  | \| \| |  |  |  |  |  |  |  |  |  |  |
| 1 | transcript:Vitvi02g00114\_t001 |  | transcript:Vitvi15g01003\_t001 |  |  |  |  |  |  |  |  |  |  |
| 1 | transcript:Vitvi02g04017\_t001 |  | \| \| |  |  |  |  |  |  |  |  |  |  |
| 1 | transcript:Vitvi02g00115\_t001 |  | \| \| |  |  |  |  |  |  |  |  |  |  |
| 1 | transcript:Vitvi02g00116\_t001 |  | \| \| |  |  |  |  |  |  |  |  |  |  |
| 1 | transcript:Vitvi02g01338\_t001 |  | \| \| |  |  |  |  |  |  |  |  |  |  |
| 1 | transcript:Vitvi02g00117\_t001 |  | \| \| |  |  |  |  |  |  |  |  |  |  |
| 1 | transcript:Vitvi02g00118\_t003 |  | \| \| |  |  |  |  |  |  |  |  |  |  |
| 1 | transcript:Vitvi02g00119\_t001 |  | transcript:Vitvi15g00997\_t001 |  |  |  |  |  |  |  |  |  |  |
| 1 | transcript:Vitvi02g00120\_t001 |  | \| \| |  |  |  |  |  |  |  |  |  |  |
| 1 | transcript:Vitvi02g00121\_t001 |  | \| \| |  |  |  |  |  |  |  |  |  |  |
| 1 | transcript:Vitvi02g04018\_t001 |  | \| \| |  |  |  |  |  |  |  |  |  |  |
| 1 | transcript:Vitvi02g00123\_t001 |  | \| \| |  |  |  |  |  |  |  |  |  |  |
| 1 | transcript:Vitvi02g00125\_t001 |  | \| \| |  |  |  |  |  |  |  |  |  |  |
| 1 | transcript:Vitvi02g00126\_t001 |  | \| \| |  |  |  |  |  |  |  |  |  |  |
| 1 | transcript:Vitvi02g00127\_t001 |  | \| \| |  |  |  |  |  |  |  |  |  |  |
| 1 | transcript:Vitvi02g00128\_t001 |  | \| \| |  |  |  |  |  |  |  |  |  |  |
| 1 | transcript:Vitvi02g00129\_t001 |  | \| \| |  |  |  |  |  |  |  |  |  |  |
| 1 | transcript:Vitvi02g00130\_t001 |  | transcript:Vitvi15g01614\_t001 |  |  |  |  |  |  |  |  |  |  |
| 0 | transcript:Vitvi02g00131\_t001 |  |  |  |  |  |  |  |  |  |  |  |  |
| 0 | transcript:Vitvi02g01339\_t001 |  |  |  |  |  |  |  |  |  |  |  |  |
| 0 | transcript:Vitvi02g04019\_t001 |  |  |  |  |  |  |  |  |  |  |  |  |
| 0 | transcript:Vitvi02g00132\_t001 |  |  |  |  |  |  |  |  |  |  |  |  |
| 0 | transcript:Vitvi02g00133\_t001 |  |  |  |  |  |  |  |  |  |  |  |  |
| 0 | transcript:Vitvi02g04020\_t001 |  |  |  |  |  |  |  |  |  |  |  |  |
| 0 | transcript:Vitvi02g00135\_t001 |  |  |  |  |  |  |  |  |  |  |  |  |
| 0 | transcript:Vitvi02g04021\_t001 |  |  |  |  |  |  |  |  |  |  |  |  |
| 0 | transcript:Vitvi02g04022\_t001 |  |  |  |  |  |  |  |  |  |  |  |  |
| 0 | transcript:Vitvi02g04023\_t001 |  |  |  |  |  |  |  |  |  |  |  |  |
| 0 | transcript:Vitvi02g00136\_t001 |  |  |  |  |  |  |  |  |  |  |  |  |
| 0 | transcript:Vitvi02g00137\_t001 |  |  |  |  |  |  |  |  |  |  |  |  |
| 0 | transcript:Vitvi02g00138\_t001 |  |  |  |  |  |  |  |  |  |  |  |  |
| 0 | transcript:Vitvi02g00139\_t001 |  |  |  |  |  |  |  |  |  |  |  |  |
| 0 | transcript:Vitvi02g00140\_t001 |  |  |  |  |  |  |  |  |  |  |  |  |
| 0 | transcript:Vitvi02g04024\_t001 |  |  |  |  |  |  |  |  |  |  |  |  |
| 0 | transcript:Vitvi02g00141\_t001 |  |  |  |  |  |  |  |  |  |  |  |  |
| 0 | transcript:Vitvi02g00143\_t001 |  |  |  |  |  |  |  |  |  |  |  |  |
| 0 | transcript:Vitvi02g00144\_t001 |  |  |  |  |  |  |  |  |  |  |  |  |
| 0 | transcript:Vitvi02g00145\_t001 |  |  |  |  |  |  |  |  |  |  |  |  |
| 0 | transcript:Vitvi02g00146\_t003 |  |  |  |  |  |  |  |  |  |  |  |  |
| 0 | transcript:Vitvi02g00147\_t001 |  |  |  |  |  |  |  |  |  |  |  |  |
| 0 | transcript:Vitvi02g00149\_t001 |  |  |  |  |  |  |  |  |  |  |  |  |
| 0 | transcript:Vitvi02g00151\_t001 |  |  |  |  |  |  |  |  |  |  |  |  |
| 0 | transcript:Vitvi02g00152\_t001 |  |  |  |  |  |  |  |  |  |  |  |  |
| 0 | transcript:Vitvi02g01340\_t001 |  |  |  |  |  |  |  |  |  |  |  |  |
| 0 | transcript:Vitvi02g04025\_t001 |  |  |  |  |  |  |  |  |  |  |  |  |
| 0 | transcript:Vitvi02g00153\_t001 |  |  |  |  |  |  |  |  |  |  |  |  |
| 0 | transcript:Vitvi02g00155\_t001 |  |  |  |  |  |  |  |  |  |  |  |  |
| 0 | transcript:Vitvi02g00156\_t001 |  |  |  |  |  |  |  |  |  |  |  |  |
| 0 | transcript:Vitvi02g00157\_t001 |  |  |  |  |  |  |  |  |  |  |  |  |
| 0 | transcript:Vitvi02g00158\_t001 |  |  |  |  |  |  |  |  |  |  |  |  |
| 0 | transcript:Vitvi02g00159\_t001 |  |  |  |  |  |  |  |  |  |  |  |  |
| 0 | transcript:Vitvi02g00160\_t001 |  |  |  |  |  |  |  |  |  |  |  |  |
| 0 | transcript:Vitvi02g00161\_t001 |  |  |  |  |  |  |  |  |  |  |  |  |
| 0 | transcript:Vitvi02g00162\_t001 |  |  |  |  |  |  |  |  |  |  |  |  |
| 0 | transcript:Vitvi02g04026\_t001 |  |  |  |  |  |  |  |  |  |  |  |  |
| 0 | transcript:Vitvi02g00163\_t001 |  |  |  |  |  |  |  |  |  |  |  |  |
| 0 | transcript:Vitvi02g04027\_t001 |  |  |  |  |  |  |  |  |  |  |  |  |
| 0 | transcript:Vitvi02g01341\_t001 |  |  |  |  |  |  |  |  |  |  |  |  |
| 0 | transcript:Vitvi02g04028\_t001 |  |  |  |  |  |  |  |  |  |  |  |  |
| 0 | transcript:Vitvi02g01343\_t001 |  |  |  |  |  |  |  |  |  |  |  |  |
| 0 | transcript:Vitvi02g04029\_t001 |  |  |  |  |  |  |  |  |  |  |  |  |
| 0 | transcript:Vitvi02g01344\_t001 |  |  |  |  |  |  |  |  |  |  |  |  |
| 0 | transcript:Vitvi02g04030\_t001 |  |  |  |  |  |  |  |  |  |  |  |  |
| 0 | transcript:Vitvi02g01353\_t001 |  |  |  |  |  |  |  |  |  |  |  |  |
| 0 | transcript:Vitvi02g04031\_t001 |  |  |  |  |  |  |  |  |  |  |  |  |
| 0 | transcript:Vitvi02g01347\_t001 |  |  |  |  |  |  |  |  |  |  |  |  |
| 0 | transcript:Vitvi02g01348\_t002 |  |  |  |  |  |  |  |  |  |  |  |  |
| 0 | transcript:Vitvi02g04032\_t001 |  |  |  |  |  |  |  |  |  |  |  |  |
| 0 | transcript:Vitvi02g04033\_t001 |  |  |  |  |  |  |  |  |  |  |  |  |
| 0 | transcript:Vitvi02g01349\_t001 |  |  |  |  |  |  |  |  |  |  |  |  |
| 0 | transcript:Vitvi02g01350\_t001 |  |  |  |  |  |  |  |  |  |  |  |  |
| 0 | transcript:Vitvi02g04034\_t001 |  |  |  |  |  |  |  |  |  |  |  |  |
| 0 | transcript:Vitvi02g04035\_t001 |  |  |  |  |  |  |  |  |  |  |  |  |
| 0 | transcript:Vitvi02g04036\_t001 |  |  |  |  |  |  |  |  |  |  |  |  |
| 0 | transcript:Vitvi02g00167\_t001 |  |  |  |  |  |  |  |  |  |  |  |  |
| 0 | transcript:Vitvi02g04037\_t001 |  |  |  |  |  |  |  |  |  |  |  |  |
| 0 | transcript:Vitvi02g04038\_t001 |  |  |  |  |  |  |  |  |  |  |  |  |
| 0 | transcript:Vitvi02g01355\_t004 |  |  |  |  |  |  |  |  |  |  |  |  |
| 0 | transcript:Vitvi02g04039\_t001 |  |  |  |  |  |  |  |  |  |  |  |  |
| 0 | transcript:Vitvi02g00174\_t001 |  |  |  |  |  |  |  |  |  |  |  |  |
| 0 | transcript:Vitvi02g01356\_t001 |  |  |  |  |  |  |  |  |  |  |  |  |
| 0 | transcript:Vitvi02g00175\_t001 |  |  |  |  |  |  |  |  |  |  |  |  |
| 0 | transcript:Vitvi02g00176\_t001 |  |  |  |  |  |  |  |  |  |  |  |  |
| 1 | transcript:Vitvi02g00177\_t001 |  | transcript:Vitvi16g01401\_t001.2.6037826c |  |  |  |  |  |  |  |  |  |  |
| 1 | transcript:Vitvi02g00178\_t001 |  | \| \| |  |  |  |  |  |  |  |  |  |  |
| 1 | transcript:Vitvi02g00179\_t001 |  | \| \| |  |  |  |  |  |  |  |  |  |  |
| 1 | transcript:Vitvi02g00180\_t001 |  | \| \| |  |  |  |  |  |  |  |  |  |  |
| 1 | transcript:Vitvi02g00181\_t001 |  | \| \| |  |  |  |  |  |  |  |  |  |  |
| 1 | transcript:Vitvi02g04040\_t001 |  | \| \| |  |  |  |  |  |  |  |  |  |  |
| 2 | transcript:Vitvi02g00182\_t001 |  | \| \| |  | transcript:Vitvi15g00943\_t001 |  |  |  |  |  |  |  |  |
| 2 | transcript:Vitvi02g04041\_t001 |  | \| \| |  | \| \| |  |  |  |  |  |  |  |  |
| 2 | transcript:Vitvi02g01358\_t001 |  | \| \| |  | \| \| |  |  |  |  |  |  |  |  |
| 2 | transcript:Vitvi02g01359\_t001 |  | \| \| |  | \| \| |  |  |  |  |  |  |  |  |
| 2 | transcript:Vitvi02g00184\_t001 |  | \| \| |  | \| \| |  |  |  |  |  |  |  |  |
| 2 | transcript:Vitvi02g00186\_t001 |  | \| \| |  | \| \| |  |  |  |  |  |  |  |  |
| 2 | transcript:Vitvi02g00187\_t001 |  | transcript:Vitvi16g01391\_t001 |  | \| \| |  |  |  |  |  |  |  |  |
| 2 | transcript:Vitvi02g00188\_t001 |  | \| \| |  | \| \| |  |  |  |  |  |  |  |  |
| 2 | transcript:Vitvi02g04042\_t001 |  | \| \| |  | \| \| |  |  |  |  |  |  |  |  |
| 2 | transcript:Vitvi02g00189\_t001 |  | \| \| |  | \| \| |  |  |  |  |  |  |  |  |
| 2 | transcript:Vitvi02g04043\_t001 |  | \| \| |  | \| \| |  |  |  |  |  |  |  |  |
| 2 | transcript:Vitvi02g04044\_t001 |  | \| \| |  | \| \| |  |  |  |  |  |  |  |  |
| 2 | transcript:Vitvi02g00191\_t001 |  | \| \| |  | \| \| |  |  |  |  |  |  |  |  |
| 2 | transcript:Vitvi02g01360\_t001 |  | \| \| |  | \| \| |  |  |  |  |  |  |  |  |
| 2 | transcript:Vitvi02g00192\_t001 |  | \| \| |  | \| \| |  |  |  |  |  |  |  |  |
| 2 | transcript:Vitvi02g00194\_t001 |  | \| \| |  | \| \| |  |  |  |  |  |  |  |  |
| 2 | transcript:Vitvi02g00195\_t001 |  | \| \| |  | transcript:Vitvi15g00938\_t001 |  |  |  |  |  |  |  |  |
| 2 | transcript:Vitvi02g04045\_t001 |  | \| \| |  | \| \| |  |  |  |  |  |  |  |  |
| 2 | transcript:Vitvi02g00197\_t001 |  | \| \| |  | \| \| |  |  |  |  |  |  |  |  |
| 2 | transcript:Vitvi02g04046\_t001 |  | \| \| |  | \| \| |  |  |  |  |  |  |  |  |
| 2 | transcript:Vitvi02g00198\_t001 |  | transcript:Vitvi16g01387\_t001 |  | transcript:Vitvi15g00937\_t001 |  |  |  |  |  |  |  |  |
| 2 | transcript:Vitvi02g00199\_t001 |  | transcript:Vitvi16g01384\_t001 |  | transcript:Vitvi15g00936\_t001 |  |  |  |  |  |  |  |  |
| 2 | transcript:Vitvi02g00200\_t001 |  | \| \| |  | transcript:Vitvi15g00935\_t001 |  |  |  |  |  |  |  |  |
| 2 | transcript:Vitvi02g00201\_t001 |  | \| \| |  | \| \| |  |  |  |  |  |  |  |  |
| 2 | transcript:Vitvi02g01361\_t001 |  | \| \| |  | \| \| |  |  |  |  |  |  |  |  |
| 2 | transcript:Vitvi02g01363\_t001 |  | \| \| |  | \| \| |  |  |  |  |  |  |  |  |
| 2 | transcript:Vitvi02g04047\_t001 |  | \| \| |  | \| \| |  |  |  |  |  |  |  |  |
| 2 | transcript:Vitvi02g00202\_t001 |  | \| \| |  | \| \| |  |  |  |  |  |  |  |  |
| 2 | transcript:Vitvi02g00203\_t001 |  | \| \| |  | transcript:Vitvi15g00934\_t001 |  |  |  |  |  |  |  |  |
| 2 | transcript:Vitvi02g00204\_t001 |  | \| \| |  | \| \| |  |  |  |  |  |  |  |  |
| 2 | transcript:Vitvi02g00205\_t001 |  | \| \| |  | \| \| |  |  |  |  |  |  |  |  |
| 2 | transcript:Vitvi02g00206\_t001 |  | \| \| |  | \| \| |  |  |  |  |  |  |  |  |
| 2 | transcript:Vitvi02g04048\_t001 |  | \| \| |  | \| \| |  |  |  |  |  |  |  |  |
| 2 | transcript:Vitvi02g00207\_t001 |  | \| \| |  | transcript:Vitvi15g00931\_t002 |  |  |  |  |  |  |  |  |
| 2 | transcript:Vitvi02g00208\_t002 |  | transcript:Vitvi16g04515\_t001 |  | transcript:Vitvi15g00929\_t001 |  |  |  |  |  |  |  |  |
| 1 | transcript:Vitvi02g00209\_t001 |  | transcript:Vitvi16g04514\_t001 |  |  |  |  |  |  |  |  |  |  |
| 1 | transcript:Vitvi02g00210\_t001 |  | \| \| |  |  |  |  |  |  |  |  |  |  |
| 1 | transcript:Vitvi02g00211\_t001 |  | \| \| |  |  |  |  |  |  |  |  |  |  |
| 1 | transcript:Vitvi02g00212\_t001 |  | transcript:Vitvi16g02074\_t001 |  |  |  |  |  |  |  |  |  |  |
| 1 | transcript:Vitvi02g00213\_t001 |  | \| \| |  |  |  |  |  |  |  |  |  |  |
| 1 | transcript:Vitvi02g00214\_t001 |  | \| \| |  |  |  |  |  |  |  |  |  |  |
| 1 | transcript:Vitvi02g04049\_t001 |  | \| \| |  |  |  |  |  |  |  |  |  |  |
| 1 | transcript:Vitvi02g01364\_t001 |  | \| \| |  |  |  |  |  |  |  |  |  |  |
| 1 | transcript:Vitvi02g00218\_t003 |  | \| \| |  |  |  |  |  |  |  |  |  |  |
| 1 | transcript:Vitvi02g00219\_t001 |  | \| \| |  |  |  |  |  |  |  |  |  |  |
| 1 | transcript:Vitvi02g01365\_t002 |  | transcript:Vitvi16g01371\_t001 |  |  |  |  |  |  |  |  |  |  |
| 1 | transcript:Vitvi02g04050\_t001 |  | \| \| |  |  |  |  |  |  |  |  |  |  |
| 1 | transcript:Vitvi02g00221\_t001 |  | \| \| |  |  |  |  |  |  |  |  |  |  |
| 1 | transcript:Vitvi02g00222\_t004 |  | transcript:Vitvi16g01366\_t002 |  |  |  |  |  |  |  |  |  |  |
| 1 | transcript:Vitvi02g00223\_t001 |  | \| \| |  |  |  |  |  |  |  |  |  |  |
| 1 | transcript:Vitvi02g00224\_t002 |  | \| \| |  |  |  |  |  |  |  |  |  |  |
| 1 | transcript:Vitvi02g00225\_t001 |  | \| \| |  |  |  |  |  |  |  |  |  |  |
| 2 | transcript:Vitvi02g00226\_t001 |  | \| \| |  | transcript:Vitvi15g00915\_t001 |  |  |  |  |  |  |  |  |
| 2 | transcript:Vitvi02g00227\_t001 |  | \| \| |  | \| \| |  |  |  |  |  |  |  |  |
| 2 | transcript:Vitvi02g00228\_t001 |  | transcript:Vitvi16g01362\_t001 |  | transcript:Vitvi15g00912\_t001 |  |  |  |  |  |  |  |  |
| 2 | transcript:Vitvi02g00230\_t001 |  | \| \| |  | \| \| |  |  |  |  |  |  |  |  |
| 2 | transcript:Vitvi02g04051\_t001 |  | \| \| |  | \| \| |  |  |  |  |  |  |  |  |
| 2 | transcript:Vitvi02g00231\_t001 |  | \| \| |  | transcript:Vitvi15g00906\_t001 |  |  |  |  |  |  |  |  |
| 2 | transcript:Vitvi02g00232\_t001 |  | \| \| |  | \| \| |  |  |  |  |  |  |  |  |
| 2 | transcript:Vitvi02g00234\_t001 |  | \| \| |  | \| \| |  |  |  |  |  |  |  |  |
| 2 | transcript:Vitvi02g00235\_t001 |  | \| \| |  | \| \| |  |  |  |  |  |  |  |  |
| 2 | transcript:Vitvi02g00236\_t001 |  | \| \| |  | \| \| |  |  |  |  |  |  |  |  |
| 2 | transcript:Vitvi02g00237\_t001 |  | \| \| |  | \| \| |  |  |  |  |  |  |  |  |
| 2 | transcript:Vitvi02g00238\_t001 |  | \| \| |  | \| \| |  |  |  |  |  |  |  |  |
| 2 | transcript:Vitvi02g04052\_t001 |  | \| \| |  | \| \| |  |  |  |  |  |  |  |  |
| 2 | transcript:Vitvi02g00239\_t001 |  | transcript:Vitvi16g01354\_t001 |  | \| \| |  |  |  |  |  |  |  |  |
| 2 | transcript:Vitvi02g00240\_t001 |  | transcript:Vitvi16g01353\_t001 |  | transcript:Vitvi15g00893\_t001 |  |  |  |  |  |  |  |  |
| 2 | transcript:Vitvi02g04053\_t001 |  | \| \| |  | \| \| |  |  |  |  |  |  |  |  |
| 2 | transcript:Vitvi02g00241\_t001 |  | \| \| |  | \| \| |  |  |  |  |  |  |  |  |
| 2 | transcript:Vitvi02g04054\_t001 |  | \| \| |  | \| \| |  |  |  |  |  |  |  |  |
| 2 | transcript:Vitvi02g00242\_t001 |  | \| \| |  | transcript:Vitvi15g00889\_t001 |  |  |  |  |  |  |  |  |
| 2 | transcript:Vitvi02g00243\_t001 |  | \| \| |  | transcript:Vitvi15g00888\_t001 |  |  |  |  |  |  |  |  |
| 2 | transcript:Vitvi02g04055\_t001 |  | transcript:Vitvi16g02070\_t001 |  | \| \| |  |  |  |  |  |  |  |  |
| 2 | transcript:Vitvi02g00244\_t001 |  | \| \| |  | \| \| |  |  |  |  |  |  |  |  |
| 2 | transcript:Vitvi02g00245\_t001 |  | transcript:Vitvi16g01350\_t001 |  | \| \| |  |  |  |  |  |  |  |  |
| 2 | transcript:Vitvi02g00246\_t001 |  | \| \| |  | \| \| |  |  |  |  |  |  |  |  |
| 2 | transcript:Vitvi02g00247\_t001 |  | \| \| |  | \| \| |  |  |  |  |  |  |  |  |
| 2 | transcript:Vitvi02g00248\_t001 |  | \| \| |  | \| \| |  |  |  |  |  |  |  |  |
| 2 | transcript:Vitvi02g00249\_t001 |  | transcript:Vitvi16g02068\_t001 |  | transcript:Vitvi15g04470\_t001 |  |  |  |  |  |  |  |  |
| 2 | transcript:Vitvi02g00250\_t001 |  | \| \| |  | transcript:Vitvi15g00885\_t001 |  |  |  |  |  |  |  |  |
| 2 | transcript:Vitvi02g00251\_t001 |  | \| \| |  | \| \| |  |  |  |  |  |  |  |  |
| 2 | transcript:Vitvi02g04056\_t001 |  | transcript:Vitvi16g02067\_t001 |  | \| \| |  |  |  |  |  |  |  |  |
| 2 | transcript:Vitvi02g00252\_t001 |  | \| \| |  | transcript:Vitvi15g00883\_t001 |  |  |  |  |  |  |  |  |
| 2 | transcript:Vitvi02g00255\_t001 |  | \| \| |  | \| \| |  |  |  |  |  |  |  |  |
| 2 | transcript:Vitvi02g00256\_t001 |  | \| \| |  | \| \| |  |  |  |  |  |  |  |  |
| 2 | transcript:Vitvi02g04057\_t001 |  | \| \| |  | \| \| |  |  |  |  |  |  |  |  |
| 2 | transcript:Vitvi02g00257\_t001 |  | \| \| |  | \| \| |  |  |  |  |  |  |  |  |
| 2 | transcript:Vitvi02g00258\_t001 |  | transcript:Vitvi16g01344\_t002 |  | \| \| |  |  |  |  |  |  |  |  |
| 2 | transcript:Vitvi02g00259\_t001 |  | \| \| |  | \| \| |  |  |  |  |  |  |  |  |
| 2 | transcript:Vitvi02g00260\_t001 |  | \| \| |  | \| \| |  |  |  |  |  |  |  |  |
| 2 | transcript:Vitvi02g01368\_t001 |  | transcript:Vitvi16g02062\_t001 |  | \| \| |  |  |  |  |  |  |  |  |
| 2 | transcript:Vitvi02g00261\_t001 |  | \| \| |  | \| \| |  |  |  |  |  |  |  |  |
| 2 | transcript:Vitvi02g01369\_t001 |  | \| \| |  | transcript:Vitvi15g00877\_t001 |  |  |  |  |  |  |  |  |
| 2 | transcript:Vitvi02g00262\_t001 |  | \| \| |  | \| \| |  |  |  |  |  |  |  |  |
| 2 | transcript:Vitvi02g04058\_t001 |  | \| \| |  | \| \| |  |  |  |  |  |  |  |  |
| 2 | transcript:Vitvi02g00263\_t001 |  | \| \| |  | \| \| |  |  |  |  |  |  |  |  |
| 2 | transcript:Vitvi02g04059\_t001 |  | transcript:Vitvi16g01336\_t001 |  | \| \| |  |  |  |  |  |  |  |  |
| 2 | transcript:Vitvi02g00265\_t001 |  | \| \| |  | \| \| |  |  |  |  |  |  |  |  |
| 2 | transcript:Vitvi02g00267\_t001 |  | \| \| |  | \| \| |  |  |  |  |  |  |  |  |
| 2 | transcript:Vitvi02g00268\_t001 |  | \| \| |  | transcript:Vitvi15g00874\_t001 |  |  |  |  |  |  |  |  |
| 2 | transcript:Vitvi02g00270\_t003 |  | \| \| |  | \| \| |  |  |  |  |  |  |  |  |
| 2 | transcript:Vitvi02g00272\_t001 |  | \| \| |  | transcript:Vitvi15g00867\_t001 |  |  |  |  |  |  |  |  |
| 2 | transcript:Vitvi02g00273\_t001 |  | \| \| |  | \| \| |  |  |  |  |  |  |  |  |
| 2 | transcript:Vitvi02g00274\_t001 |  | \| \| |  | \| \| |  |  |  |  |  |  |  |  |
| 2 | transcript:Vitvi02g04060\_t001 |  | \| \| |  | \| \| |  |  |  |  |  |  |  |  |
| 2 | transcript:Vitvi02g00275\_t001 |  | \| \| |  | transcript:Vitvi15g00863\_t001 |  |  |  |  |  |  |  |  |
| 2 | transcript:Vitvi02g04061\_t001 |  | \| \| |  | \| \| |  |  |  |  |  |  |  |  |
| 2 | transcript:Vitvi02g00276\_t001 |  | \| \| |  | \| \| |  |  |  |  |  |  |  |  |
| 2 | transcript:Vitvi02g04062\_t001 |  | \| \| |  | \| \| |  |  |  |  |  |  |  |  |
| 2 | transcript:Vitvi02g00277\_t001 |  | transcript:Vitvi16g01327\_t002 |  | transcript:Vitvi15g01539\_t001 |  |  |  |  |  |  |  |  |
| 0 | transcript:Vitvi02g04063\_t001 |  |  |  |  |  |  |  |  |  |  |  |  |
| 0 | transcript:Vitvi02g00278\_t001 |  |  |  |  |  |  |  |  |  |  |  |  |
| 0 | transcript:Vitvi02g01371\_t001 |  |  |  |  |  |  |  |  |  |  |  |  |
| 0 | transcript:Vitvi02g00279\_t001 |  |  |  |  |  |  |  |  |  |  |  |  |
| 0 | transcript:Vitvi02g00280\_t001 |  |  |  |  |  |  |  |  |  |  |  |  |
| 0 | transcript:Vitvi02g04064\_t001 |  |  |  |  |  |  |  |  |  |  |  |  |
| 0 | transcript:Vitvi02g00282\_t001 |  |  |  |  |  |  |  |  |  |  |  |  |
| 0 | transcript:Vitvi02g00283\_t001 |  |  |  |  |  |  |  |  |  |  |  |  |
| 0 | transcript:Vitvi02g01372\_t001 |  |  |  |  |  |  |  |  |  |  |  |  |
| 0 | transcript:Vitvi02g01373\_t001 |  |  |  |  |  |  |  |  |  |  |  |  |
| 0 | transcript:Vitvi02g01374\_t001 |  |  |  |  |  |  |  |  |  |  |  |  |
| 0 | transcript:Vitvi02g01375\_t002 |  |  |  |  |  |  |  |  |  |  |  |  |
| 0 | transcript:Vitvi02g00284\_t001 |  |  |  |  |  |  |  |  |  |  |  |  |
| 0 | transcript:Vitvi02g04065\_t001 |  |  |  |  |  |  |  |  |  |  |  |  |
| 0 | transcript:Vitvi02g00286\_t001 |  |  |  |  |  |  |  |  |  |  |  |  |
| 0 | transcript:Vitvi02g00287\_t001 |  |  |  |  |  |  |  |  |  |  |  |  |
| 0 | transcript:Vitvi02g00289\_t001 |  |  |  |  |  |  |  |  |  |  |  |  |
| 0 | transcript:Vitvi02g00290\_t001 |  |  |  |  |  |  |  |  |  |  |  |  |
| 0 | transcript:Vitvi02g00291\_t001 |  |  |  |  |  |  |  |  |  |  |  |  |
| 0 | transcript:Vitvi02g04066\_t001 |  |  |  |  |  |  |  |  |  |  |  |  |
| 0 | transcript:Vitvi02g00293\_t001 |  |  |  |  |  |  |  |  |  |  |  |  |
| 0 | transcript:Vitvi02g04067\_t001 |  |  |  |  |  |  |  |  |  |  |  |  |
| 0 | transcript:Vitvi02g04068\_t001 |  |  |  |  |  |  |  |  |  |  |  |  |
| 0 | transcript:Vitvi02g00295\_t001 |  |  |  |  |  |  |  |  |  |  |  |  |
| 0 | transcript:Vitvi02g00296\_t001 |  |  |  |  |  |  |  |  |  |  |  |  |
| 1 | transcript:Vitvi02g00297\_t001 |  | transcript:Vitvi16g01074\_t001 |  |  |  |  |  |  |  |  |  |  |
| 1 | transcript:Vitvi02g00298\_t001 |  | \| \| |  |  |  |  |  |  |  |  |  |  |
| 1 | transcript:Vitvi02g00301\_t001 |  | transcript:Vitvi16g01073\_t001 |  |  |  |  |  |  |  |  |  |  |
| 2 | transcript:Vitvi02g00303\_t001 |  | \| \| |  | transcript:Vitvi15g01106\_t001 |  |  |  |  |  |  |  |  |
| 2 | transcript:Vitvi02g00304\_t001 |  | \| \| |  | \| \| |  |  |  |  |  |  |  |  |
| 2 | transcript:Vitvi02g04069\_t001 |  | \| \| |  | \| \| |  |  |  |  |  |  |  |  |
| 2 | transcript:Vitvi02g00308\_t001 |  | transcript:Vitvi16g01071\_t001 |  | \| \| |  |  |  |  |  |  |  |  |
| 2 | transcript:Vitvi02g00309\_t001 |  | transcript:Vitvi16g04376\_t001 |  | \| \| |  |  |  |  |  |  |  |  |
| 2 | transcript:Vitvi02g00310\_t001 |  | \| \| |  | transcript:Vitvi15g01110\_t002 |  |  |  |  |  |  |  |  |
| 2 | transcript:Vitvi02g00311\_t001 |  | transcript:Vitvi16g01051\_t001 |  | transcript:Vitvi15g01114\_t001 |  |  |  |  |  |  |  |  |
| 2 | transcript:Vitvi02g04070\_t001 |  | \| \| |  | \| \| |  |  |  |  |  |  |  |  |
| 2 | transcript:Vitvi02g01378\_t001 |  | \| \| |  | \| \| |  |  |  |  |  |  |  |  |
| 2 | transcript:Vitvi02g00313\_t001 |  | \| \| |  | \| \| |  |  |  |  |  |  |  |  |
| 2 | transcript:Vitvi02g00314\_t002 |  | \| \| |  | transcript:Vitvi15g01117\_t002 |  |  |  |  |  |  |  |  |
| 2 | transcript:Vitvi02g00315\_t001 |  | \| \| |  | \| \| |  |  |  |  |  |  |  |  |
| 2 | transcript:Vitvi02g00316\_t001 |  | \| \| |  | transcript:Vitvi15g01123\_t001 |  |  |  |  |  |  |  |  |
| 2 | transcript:Vitvi02g00317\_t001 |  | \| \| |  | transcript:Vitvi15g01124\_t001 |  |  |  |  |  |  |  |  |
| 1 | transcript:Vitvi02g01379\_t001 |  | \| \| |  |  |  |  |  |  |  |  |  |  |
| 1 | transcript:Vitvi02g00318\_t001 |  | \| \| |  |  |  |  |  |  |  |  |  |  |
| 1 | transcript:Vitvi02g04071\_t001 |  | \| \| |  |  |  |  |  |  |  |  |  |  |
| 1 | transcript:Vitvi02g00319\_t001 |  | \| \| |  |  |  |  |  |  |  |  |  |  |
| 1 | transcript:Vitvi02g00320\_t001 |  | \| \| |  |  |  |  |  |  |  |  |  |  |
| 1 | transcript:Vitvi02g04072\_t001 |  | \| \| |  |  |  |  |  |  |  |  |  |  |
| 1 | transcript:Vitvi02g04073\_t001 |  | \| \| |  |  |  |  |  |  |  |  |  |  |
| 1 | transcript:Vitvi02g00324\_t001 |  | \| \| |  |  |  |  |  |  |  |  |  |  |
| 1 | transcript:Vitvi02g00325\_t001 |  | \| \| |  |  |  |  |  |  |  |  |  |  |
| 1 | transcript:Vitvi02g00326\_t002 |  | \| \| |  |  |  |  |  |  |  |  |  |  |
| 1 | transcript:Vitvi02g00327\_t001 |  | transcript:Vitvi16g04364\_t001 |  |  |  |  |  |  |  |  |  |  |
| 2 | transcript:Vitvi02g00328\_t003 |  | \| \| |  | transcript:Vitvi15g01144\_t001 |  |  |  |  |  |  |  |  |
| 2 | transcript:Vitvi02g00329\_t001 |  | \| \| |  | \| \| |  |  |  |  |  |  |  |  |
| 2 | transcript:Vitvi02g00330\_t001 |  | \| \| |  | \| \| |  |  |  |  |  |  |  |  |
| 2 | transcript:Vitvi02g04074\_t001 |  | \| \| |  | \| \| |  |  |  |  |  |  |  |  |
| 2 | transcript:Vitvi02g01380\_t001 |  | \| \| |  | \| \| |  |  |  |  |  |  |  |  |
| 2 | transcript:Vitvi02g00331\_t001 |  | \| \| |  | transcript:Vitvi15g01145\_t001 |  |  |  |  |  |  |  |  |
| 2 | transcript:Vitvi02g00332\_t001 |  | \| \| |  | \| \| |  |  |  |  |  |  |  |  |
| 2 | transcript:Vitvi02g00333\_t001 |  | \| \| |  | \| \| |  |  |  |  |  |  |  |  |
| 2 | transcript:Vitvi02g00334\_t001 |  | \| \| |  | \| \| |  |  |  |  |  |  |  |  |
| 2 | transcript:Vitvi02g01381\_t001 |  | \| \| |  | transcript:Vitvi15g01147\_t001 |  |  |  |  |  |  |  |  |
| 2 | transcript:Vitvi02g04075\_t001 |  | \| \| |  | \| \| |  |  |  |  |  |  |  |  |
| 2 | transcript:Vitvi02g00335\_t001 |  | \| \| |  | \| \| |  |  |  |  |  |  |  |  |
| 2 | transcript:Vitvi02g00337\_t001 |  | \| \| |  | \| \| |  |  |  |  |  |  |  |  |
| 2 | transcript:Vitvi02g01382\_t001 |  | \| \| |  | \| \| |  |  |  |  |  |  |  |  |
| 2 | transcript:Vitvi02g01383\_t001 |  | \| \| |  | \| \| |  |  |  |  |  |  |  |  |
| 2 | transcript:Vitvi02g00338\_t001 |  | \| \| |  | \| \| |  |  |  |  |  |  |  |  |
| 2 | transcript:Vitvi02g00339\_t001 |  | \| \| |  | \| \| |  |  |  |  |  |  |  |  |
| 2 | transcript:Vitvi02g00340\_t001 |  | transcript:Vitvi16g01029\_t001 |  | \| \| |  |  |  |  |  |  |  |  |
| 2 | transcript:Vitvi02g00341\_t001 |  | \| \| |  | \| \| |  |  |  |  |  |  |  |  |
| 2 | transcript:Vitvi02g00342\_t001 |  | \| \| |  | \| \| |  |  |  |  |  |  |  |  |
| 2 | transcript:Vitvi02g01384\_t001 |  | transcript:Vitvi16g01024\_t001 |  | transcript:Vitvi15g01683\_t001 |  |  |  |  |  |  |  |  |
| 2 | transcript:Vitvi02g04076\_t001 |  | \| \| |  | \| \| |  |  |  |  |  |  |  |  |
| 2 | transcript:Vitvi02g00344\_t001 |  | \| \| |  | \| \| |  |  |  |  |  |  |  |  |
| 2 | transcript:Vitvi02g04077\_t001 |  | \| \| |  | \| \| |  |  |  |  |  |  |  |  |
| 2 | transcript:Vitvi02g01385\_t001 |  | \| \| |  | \| \| |  |  |  |  |  |  |  |  |
| 2 | transcript:Vitvi02g00345\_t001 |  | \| \| |  | \| \| |  |  |  |  |  |  |  |  |
| 2 | transcript:Vitvi02g00346\_t001 |  | transcript:Vitvi16g01874\_t001 |  | \| \| |  |  |  |  |  |  |  |  |
| 2 | transcript:Vitvi02g00347\_t001 |  | \| \| |  | \| \| |  |  |  |  |  |  |  |  |
| 2 | transcript:Vitvi02g04078\_t001 |  | \| \| |  | \| \| |  |  |  |  |  |  |  |  |
| 2 | transcript:Vitvi02g00348\_t001 |  | transcript:Vitvi16g01018\_t001 |  | transcript:Vitvi15g01160\_t001 |  |  |  |  |  |  |  |  |
| 2 | transcript:Vitvi02g00349\_t001 |  | \| \| |  | transcript:Vitvi15g01162\_t002 |  |  |  |  |  |  |  |  |
| 2 | transcript:Vitvi02g04079\_t001 |  | \| \| |  | \| \| |  |  |  |  |  |  |  |  |
| 2 | transcript:Vitvi02g04080\_t001 |  | \| \| |  | \| \| |  |  |  |  |  |  |  |  |
| 2 | transcript:Vitvi02g04081\_t001 |  | \| \| |  | \| \| |  |  |  |  |  |  |  |  |
| 2 | transcript:Vitvi02g00350\_t001 |  | \| \| |  | \| \| |  |  |  |  |  |  |  |  |
| 2 | transcript:Vitvi02g04082\_t001 |  | \| \| |  | \| \| |  |  |  |  |  |  |  |  |
| 2 | transcript:Vitvi02g04083\_t001 |  | \| \| |  | \| \| |  |  |  |  |  |  |  |  |
| 2 | transcript:Vitvi02g04084\_t001 |  | \| \| |  | \| \| |  |  |  |  |  |  |  |  |
| 2 | transcript:Vitvi02g00358\_t001 |  | \| \| |  | \| \| |  |  |  |  |  |  |  |  |
| 2 | transcript:Vitvi02g00360\_t001 |  | \| \| |  | \| \| |  |  |  |  |  |  |  |  |
| 2 | transcript:Vitvi02g04085\_t001 |  | \| \| |  | \| \| |  |  |  |  |  |  |  |  |
| 2 | transcript:Vitvi02g01392\_t001 |  | \| \| |  | \| \| |  |  |  |  |  |  |  |  |
| 2 | transcript:Vitvi02g00363\_t001 |  | \| \| |  | \| \| |  |  |  |  |  |  |  |  |
| 2 | transcript:Vitvi02g00364\_t001 |  | \| \| |  | \| \| |  |  |  |  |  |  |  |  |
| 2 | transcript:Vitvi02g04086\_t001 |  | \| \| |  | \| \| |  |  |  |  |  |  |  |  |
| 2 | transcript:Vitvi02g00365\_t001 |  | \| \| |  | \| \| |  |  |  |  |  |  |  |  |
| 2 | transcript:Vitvi02g01393\_t001 |  | \| \| |  | \| \| |  |  |  |  |  |  |  |  |
| 2 | transcript:Vitvi02g00366\_t001 |  | \| \| |  | \| \| |  |  |  |  |  |  |  |  |
| 2 | transcript:Vitvi02g00367\_t001 |  | \| \| |  | \| \| |  |  |  |  |  |  |  |  |
| 2 | transcript:Vitvi02g04087\_t001 |  | \| \| |  | \| \| |  |  |  |  |  |  |  |  |
| 2 | transcript:Vitvi02g00368\_t001 |  | \| \| |  | transcript:Vitvi15g01171\_t001 |  |  |  |  |  |  |  |  |
| 1 | transcript:Vitvi02g00370\_t001 |  | \| \| |  |  |  |  |  |  |  |  |  |  |
| 2 | transcript:Vitvi02g01397\_t001 |  | \| \| |  | transcript:Vitvi16g00961\_t001 |  |  |  |  |  |  |  |  |
| 2 | transcript:Vitvi02g01398\_t001 |  | transcript:Vitvi16g01872\_t001 |  | \| \| |  |  |  |  |  |  |  |  |
| 2 | transcript:Vitvi02g04088\_t001 |  | \| \| |  | \| \| |  |  |  |  |  |  |  |  |
| 2 | transcript:Vitvi02g00373\_t001 |  | \| \| |  | \| \| |  |  |  |  |  |  |  |  |
| 2 | transcript:Vitvi02g00374\_t001 |  | \| \| |  | \| \| |  |  |  |  |  |  |  |  |
| 2 | transcript:Vitvi02g00375\_t001 |  | \| \| |  | \| \| |  |  |  |  |  |  |  |  |
| 2 | transcript:Vitvi02g00377\_t001 |  | \| \| |  | transcript:Vitvi16g00966\_t001 |  |  |  |  |  |  |  |  |
| 2 | transcript:Vitvi02g00378\_t001 |  | \| \| |  | \| \| |  |  |  |  |  |  |  |  |
| 2 | transcript:Vitvi02g00379\_t001 |  | \| \| |  | \| \| |  |  |  |  |  |  |  |  |
| 2 | transcript:Vitvi02g04089\_t001 |  | \| \| |  | \| \| |  |  |  |  |  |  |  |  |
| 2 | transcript:Vitvi02g00380\_t001 |  | \| \| |  | \| \| |  |  |  |  |  |  |  |  |
| 2 | transcript:Vitvi02g04090\_t001 |  | \| \| |  | \| \| |  |  |  |  |  |  |  |  |
| 2 | transcript:Vitvi02g00381\_t001 |  | \| \| |  | transcript:Vitvi16g00970\_t001 |  |  |  |  |  |  |  |  |
| 2 | transcript:Vitvi02g00382\_t001 |  | \| \| |  | transcript:Vitvi16g00972\_t001 |  |  |  |  |  |  |  |  |
| 2 | transcript:Vitvi02g00383\_t001 |  | \| \| |  | \| \| |  |  |  |  |  |  |  |  |
| 2 | transcript:Vitvi02g00384\_t001 |  | \| \| |  | \| \| |  |  |  |  |  |  |  |  |
| 2 | transcript:Vitvi02g00385\_t001 |  | \| \| |  | \| \| |  |  |  |  |  |  |  |  |
| 2 | transcript:Vitvi02g00386\_t001 |  | \| \| |  | \| \| |  |  |  |  |  |  |  |  |
| 2 | transcript:Vitvi02g00387\_t001 |  | transcript:Vitvi16g00982\_t001 |  | transcript:Vitvi16g00982\_t001 |  |  |  |  |  |  |  |  |
| 2 | transcript:Vitvi02g00388\_t001 |  | \| \| |  | transcript:Vitvi16g01871\_t003 |  |  |  |  |  |  |  |  |
| 1 | transcript:Vitvi02g00389\_t001 |  | \| \| |  |  |  |  |  |  |  |  |  |  |
| 1 | transcript:Vitvi02g01399\_t001 |  | \| \| |  |  |  |  |  |  |  |  |  |  |
| 1 | transcript:Vitvi02g00390\_t001 |  | \| \| |  |  |  |  |  |  |  |  |  |  |
| 1 | transcript:Vitvi02g01400\_t001 |  | \| \| |  |  |  |  |  |  |  |  |  |  |
| 1 | transcript:Vitvi02g00391\_t001 |  | \| \| |  |  |  |  |  |  |  |  |  |  |
| 1 | transcript:Vitvi02g00393\_t001 |  | \| \| |  |  |  |  |  |  |  |  |  |  |
| 1 | transcript:Vitvi02g01403\_t001 |  | \| \| |  |  |  |  |  |  |  |  |  |  |
| 1 | transcript:Vitvi02g01404\_t001 |  | \| \| |  |  |  |  |  |  |  |  |  |  |
| 1 | transcript:Vitvi02g01405\_t001 |  | \| \| |  |  |  |  |  |  |  |  |  |  |
| 1 | transcript:Vitvi02g04091\_t001 |  | \| \| |  |  |  |  |  |  |  |  |  |  |
| 1 | transcript:Vitvi02g01406\_t001 |  | \| \| |  |  |  |  |  |  |  |  |  |  |
| 1 | transcript:Vitvi02g04092\_t001 |  | \| \| |  |  |  |  |  |  |  |  |  |  |
| 1 | transcript:Vitvi02g01407\_t001 |  | \| \| |  |  |  |  |  |  |  |  |  |  |
| 1 | transcript:Vitvi02g01408\_t001 |  | \| \| |  |  |  |  |  |  |  |  |  |  |
| 1 | transcript:Vitvi02g01409\_t001 |  | \| \| |  |  |  |  |  |  |  |  |  |  |
| 1 | transcript:Vitvi02g00394\_t001 |  | \| \| |  |  |  |  |  |  |  |  |  |  |
| 1 | transcript:Vitvi02g04093\_t001 |  | \| \| |  |  |  |  |  |  |  |  |  |  |
| 1 | transcript:Vitvi02g00395\_t001 |  | transcript:Vitvi16g00961\_t001 |  |  |  |  |  |  |  |  |  |  |
| 1 | transcript:Vitvi02g00396\_t001 |  | \| \| |  |  |  |  |  |  |  |  |  |  |
| 1 | transcript:Vitvi02g00397\_t001 |  | \| \| |  |  |  |  |  |  |  |  |  |  |
| 1 | transcript:Vitvi02g00398\_t001 |  | transcript:Vitvi16g00957\_t001 |  |  |  |  |  |  |  |  |  |  |
| 1 | transcript:Vitvi02g00399\_t001 |  | transcript:Vitvi16g00956\_t001 |  |  |  |  |  |  |  |  |  |  |
| 1 | transcript:Vitvi02g04094\_t001 |  | \| \| |  |  |  |  |  |  |  |  |  |  |
| 1 | transcript:Vitvi02g00402\_t001 |  | \| \| |  |  |  |  |  |  |  |  |  |  |
| 1 | transcript:Vitvi02g00403\_t001 |  | transcript:Vitvi16g00951\_t001 |  |  |  |  |  |  |  |  |  |  |
| 1 | transcript:Vitvi02g00404\_t001 |  | \| \| |  |  |  |  |  |  |  |  |  |  |
| 1 | transcript:Vitvi02g04095\_t001 |  | transcript:Vitvi16g00942\_t001 |  |  |  |  |  |  |  |  |  |  |
| 1 | transcript:Vitvi02g00407\_t001 |  | transcript:Vitvi16g04340\_t001 |  |  |  |  |  |  |  |  |  |  |
| 1 | transcript:Vitvi02g04096\_t001 |  | \| \| |  |  |  |  |  |  |  |  |  |  |
| 1 | transcript:Vitvi02g04097\_t001 |  | \| \| |  |  |  |  |  |  |  |  |  |  |
| 2 | transcript:Vitvi02g00409\_t001 |  | \| \| |  | transcript:Vitvi15g00753\_t001 |  |  |  |  |  |  |  |  |
| 2 | transcript:Vitvi02g04098\_t001 |  | \| \| |  | \| \| |  |  |  |  |  |  |  |  |
| 2 | transcript:Vitvi02g04099\_t001 |  | \| \| |  | \| \| |  |  |  |  |  |  |  |  |
| 2 | transcript:Vitvi02g00410\_t001 |  | \| \| |  | transcript:Vitvi15g00754\_t001 |  |  |  |  |  |  |  |  |
| 2 | transcript:Vitvi02g00411\_t001 |  | \| \| |  | \| \| |  |  |  |  |  |  |  |  |
| 2 | transcript:Vitvi02g00412\_t001 |  | \| \| |  | \| \| |  |  |  |  |  |  |  |  |
| 2 | transcript:Vitvi02g00413\_t002 |  | transcript:Vitvi16g00935\_t001 |  | \| \| |  |  |  |  |  |  |  |  |
| 2 | transcript:Vitvi02g00414\_t001 |  | \| \| |  | \| \| |  |  |  |  |  |  |  |  |
| 2 | transcript:Vitvi02g00415\_t001 |  | \| \| |  | transcript:Vitvi15g00759\_t001 |  |  |  |  |  |  |  |  |
| 2 | transcript:Vitvi02g00416\_t001 |  | transcript:Vitvi16g01854\_t001 |  | \| \| |  |  |  |  |  |  |  |  |
| 2 | transcript:Vitvi02g00417\_t001 |  | \| \| |  | \| \| |  |  |  |  |  |  |  |  |
| 2 | transcript:Vitvi02g00419\_t001 |  | transcript:Vitvi16g00922\_t001 |  | \| \| |  |  |  |  |  |  |  |  |
| 2 | transcript:Vitvi02g00420\_t002 |  | transcript:Vitvi16g00919\_t001 |  | \| \| |  |  |  |  |  |  |  |  |
| 2 | transcript:Vitvi02g04100\_t001 |  | \| \| |  | \| \| |  |  |  |  |  |  |  |  |
| 2 | transcript:Vitvi02g00421\_t001 |  | transcript:Vitvi16g01846\_t001 |  | transcript:Vitvi15g00765\_t001 |  |  |  |  |  |  |  |  |
| 2 | transcript:Vitvi02g00422\_t001 |  | transcript:Vitvi16g01838\_t001 |  | \| \| |  |  |  |  |  |  |  |  |
| 2 | transcript:Vitvi02g00423\_t001 |  | \| \| |  | \| \| |  |  |  |  |  |  |  |  |
| 2 | transcript:Vitvi02g00424\_t001 |  | \| \| |  | transcript:Vitvi15g00766\_t001 |  |  |  |  |  |  |  |  |
| 2 | transcript:Vitvi02g04101\_t001 |  | \| \| |  | \| \| |  |  |  |  |  |  |  |  |
| 2 | transcript:Vitvi02g00425\_t001 |  | \| \| |  | \| \| |  |  |  |  |  |  |  |  |
| 2 | transcript:Vitvi02g00426\_t001 |  | \| \| |  | \| \| |  |  |  |  |  |  |  |  |
| 2 | transcript:Vitvi02g00427\_t001 |  | transcript:Vitvi16g00898\_t001 |  | transcript:Vitvi15g00774\_t002 |  |  |  |  |  |  |  |  |
| 2 | transcript:Vitvi02g04102\_t001 |  | \| \| |  | \| \| |  |  |  |  |  |  |  |  |
| 2 | transcript:Vitvi02g00429\_t002 |  | \| \| |  | \| \| |  |  |  |  |  |  |  |  |
| 2 | transcript:Vitvi02g00431\_t001 |  | \| \| |  | \| \| |  |  |  |  |  |  |  |  |
| 2 | transcript:Vitvi02g00432\_t001 |  | \| \| |  | \| \| |  |  |  |  |  |  |  |  |
| 2 | transcript:Vitvi02g00433\_t001 |  | \| \| |  | \| \| |  |  |  |  |  |  |  |  |
| 2 | transcript:Vitvi02g00434\_t001 |  | \| \| |  | \| \| |  |  |  |  |  |  |  |  |
| 2 | transcript:Vitvi02g04103\_t001 |  | \| \| |  | \| \| |  |  |  |  |  |  |  |  |
| 2 | transcript:Vitvi02g04104\_t001 |  | \| \| |  | \| \| |  |  |  |  |  |  |  |  |
| 2 | transcript:Vitvi02g00435\_t001 |  | \| \| |  | \| \| |  |  |  |  |  |  |  |  |
| 2 | transcript:Vitvi02g00436\_t001 |  | \| \| |  | transcript:Vitvi15g00783\_t002 |  |  |  |  |  |  |  |  |
| 1 | transcript:Vitvi02g00437\_t002 |  | \| \| |  |  |  |  |  |  |  |  |  |  |
| 1 | transcript:Vitvi02g00439\_t001 |  | transcript:Vitvi16g00882\_t001 |  |  |  |  |  |  |  |  |  |  |
| 0 | transcript:Vitvi02g00440\_t002 |  |  |  |  |  |  |  |  |  |  |  |  |
| 0 | transcript:Vitvi02g00441\_t002 |  |  |  |  |  |  |  |  |  |  |  |  |
| 0 | transcript:Vitvi02g00442\_t001 |  |  |  |  |  |  |  |  |  |  |  |  |
| 0 | transcript:Vitvi02g01411\_t001 |  |  |  |  |  |  |  |  |  |  |  |  |
| 0 | transcript:Vitvi02g01412\_t001 |  |  |  |  |  |  |  |  |  |  |  |  |
| 0 | transcript:Vitvi02g01413\_t001 |  |  |  |  |  |  |  |  |  |  |  |  |
| 0 | transcript:Vitvi02g00443\_t001 |  |  |  |  |  |  |  |  |  |  |  |  |
| 0 | transcript:Vitvi02g00444\_t001 |  |  |  |  |  |  |  |  |  |  |  |  |
| 0 | transcript:Vitvi02g04105\_t001 |  |  |  |  |  |  |  |  |  |  |  |  |
| 0 | transcript:Vitvi02g00445\_t001 |  |  |  |  |  |  |  |  |  |  |  |  |
| 0 | transcript:Vitvi02g00446\_t001 |  |  |  |  |  |  |  |  |  |  |  |  |
| 0 | transcript:Vitvi02g04106\_t001 |  |  |  |  |  |  |  |  |  |  |  |  |
| 0 | transcript:Vitvi02g04107\_t001 |  |  |  |  |  |  |  |  |  |  |  |  |
| 0 | transcript:Vitvi02g04108\_t001 |  |  |  |  |  |  |  |  |  |  |  |  |
| 0 | transcript:Vitvi02g04109\_t001 |  |  |  |  |  |  |  |  |  |  |  |  |
| 0 | transcript:Vitvi02g04110\_t001 |  |  |  |  |  |  |  |  |  |  |  |  |
| 0 | transcript:Vitvi02g01414\_t001 |  |  |  |  |  |  |  |  |  |  |  |  |
| 0 | transcript:Vitvi02g04111\_t001 |  |  |  |  |  |  |  |  |  |  |  |  |
| 0 | transcript:Vitvi02g04112\_t001 |  |  |  |  |  |  |  |  |  |  |  |  |
| 0 | transcript:Vitvi02g04113\_t001 |  |  |  |  |  |  |  |  |  |  |  |  |
| 0 | transcript:Vitvi02g00448\_t001 |  |  |  |  |  |  |  |  |  |  |  |  |
| 0 | transcript:Vitvi02g00449\_t001 |  |  |  |  |  |  |  |  |  |  |  |  |
| 0 | transcript:Vitvi02g00450\_t001 |  |  |  |  |  |  |  |  |  |  |  |  |
| 0 | transcript:Vitvi02g04114\_t001 |  |  |  |  |  |  |  |  |  |  |  |  |
| 0 | transcript:Vitvi02g00451\_t001 |  |  |  |  |  |  |  |  |  |  |  |  |
| 0 | transcript:Vitvi02g00452\_t001 |  |  |  |  |  |  |  |  |  |  |  |  |
| 0 | transcript:Vitvi02g01417\_t001 |  |  |  |  |  |  |  |  |  |  |  |  |
| 2 | transcript:Vitvi02g00454\_t001 |  | transcript:Vitvi00g04333\_t001 |  | transcript:Vitvi16g00855\_t001 |  |  |  |  |  |  |  |  |
| 2 | transcript:Vitvi02g04115\_t001 |  | transcript:Vitvi00g04334\_t001 |  | \| \| |  |  |  |  |  |  |  |  |
| 2 | transcript:Vitvi02g04116\_t001 |  | transcript:Vitvi00g04335\_t001 |  | \| \| |  |  |  |  |  |  |  |  |
| 2 | transcript:Vitvi02g04117\_t001 |  | transcript:Vitvi00g04336\_t001 |  | transcript:Vitvi16g00853\_t001 |  |  |  |  |  |  |  |  |
| 2 | transcript:Vitvi02g04118\_t001 |  | transcript:Vitvi00g04337\_t001 |  | \| \| |  |  |  |  |  |  |  |  |
| 2 | transcript:Vitvi02g04119\_t001 |  | transcript:Vitvi00g04338\_t001 |  | \| \| |  |  |  |  |  |  |  |  |
| 2 | transcript:Vitvi02g04120\_t001 |  | transcript:Vitvi00g04339\_t001 |  | \| \| |  |  |  |  |  |  |  |  |
| 2 | transcript:Vitvi02g00459\_t001 |  | transcript:Vitvi00g04340\_t001 |  | \| \| |  |  |  |  |  |  |  |  |
| 2 | transcript:Vitvi02g00460\_t002 |  | transcript:Vitvi15g00727\_t001 |  | \| \| |  |  |  |  |  |  |  |  |
| 2 | transcript:Vitvi02g00461\_t002 |  | \| \| |  | \| \| |  |  |  |  |  |  |  |  |
| 2 | transcript:Vitvi02g00462\_t001 |  | transcript:Vitvi15g00731\_t001 |  | \| \| |  |  |  |  |  |  |  |  |
| 2 | transcript:Vitvi02g00465\_t001 |  | transcript:Vitvi15g00732\_t001 |  | \| \| |  |  |  |  |  |  |  |  |
| 2 | transcript:Vitvi02g00466\_t001 |  | \| \| |  | \| \| |  |  |  |  |  |  |  |  |
| 2 | transcript:Vitvi02g04121\_t001 |  | \| \| |  | \| \| |  |  |  |  |  |  |  |  |
| 2 | transcript:Vitvi02g04122\_t001 |  | \| \| |  | \| \| |  |  |  |  |  |  |  |  |
| 2 | transcript:Vitvi02g00467\_t001 |  | \| \| |  | \| \| |  |  |  |  |  |  |  |  |
| 2 | transcript:Vitvi02g00468\_t001 |  | transcript:Vitvi15g00740\_t001 |  | transcript:Vitvi16g00835\_t001 |  |  |  |  |  |  |  |  |
| 2 | transcript:Vitvi02g04123\_t001 |  | transcript:Vitvi15g01500\_t001 |  | \| \| |  |  |  |  |  |  |  |  |
| 2 | transcript:Vitvi02g04124\_t001 |  | \| \| |  | \| \| |  |  |  |  |  |  |  |  |
| 2 | transcript:Vitvi02g00469\_t001 |  | \| \| |  | \| \| |  |  |  |  |  |  |  |  |
| 2 | transcript:Vitvi02g00470\_t001 |  | \| \| |  | \| \| |  |  |  |  |  |  |  |  |
| 2 | transcript:Vitvi02g00471\_t001 |  | transcript:Vitvi15g00741\_t001 |  | \| \| |  |  |  |  |  |  |  |  |
| 1 | transcript:Vitvi02g00472\_t001 |  |  |  | \| \| |  |  |  |  |  |  |  |  |
| 1 | transcript:Vitvi02g04125\_t001 |  |  |  | \| \| |  |  |  |  |  |  |  |  |
| 1 | transcript:Vitvi02g00474\_t001 |  |  |  | \| \| |  |  |  |  |  |  |  |  |
| 1 | transcript:Vitvi02g01423\_t001 |  |  |  | \| \| |  |  |  |  |  |  |  |  |
| 1 | transcript:Vitvi02g04126\_t001 |  |  |  | \| \| |  |  |  |  |  |  |  |  |
| 1 | transcript:Vitvi02g00475\_t001 |  |  |  | \| \| |  |  |  |  |  |  |  |  |
| 1 | transcript:Vitvi02g00476\_t002 |  |  |  | transcript:Vitvi16g00826\_t001 |  |  |  |  |  |  |  |  |
| 1 | transcript:Vitvi02g04127\_t001 |  |  |  | \| \| |  |  |  |  |  |  |  |  |
| 1 | transcript:Vitvi02g00477\_t001 |  |  |  | \| \| |  |  |  |  |  |  |  |  |
| 1 | transcript:Vitvi02g04128\_t001 |  |  |  | \| \| |  |  |  |  |  |  |  |  |
| 1 | transcript:Vitvi02g04129\_t001 |  |  |  | \| \| |  |  |  |  |  |  |  |  |
| 1 | transcript:Vitvi02g00479\_t001 |  |  |  | \| \| |  |  |  |  |  |  |  |  |
| 1 | transcript:Vitvi02g00480\_t001 |  |  |  | \| \| |  |  |  |  |  |  |  |  |
| 1 | transcript:Vitvi02g00481\_t001 |  |  |  | transcript:Vitvi16g00809\_t001 |  |  |  |  |  |  |  |  |
| 1 | transcript:Vitvi02g00482\_t001 |  |  |  | \| \| |  |  |  |  |  |  |  |  |
| 1 | transcript:Vitvi02g00483\_t001 |  |  |  | transcript:Vitvi16g00808\_t001 |  |  |  |  |  |  |  |  |
| 1 | transcript:Vitvi02g00485\_t001 |  |  |  | \| \| |  |  |  |  |  |  |  |  |
| 1 | transcript:Vitvi02g00486\_t001 |  |  |  | \| \| |  |  |  |  |  |  |  |  |
| 1 | transcript:Vitvi02g00487\_t001 |  |  |  | \| \| |  |  |  |  |  |  |  |  |
| 1 | transcript:Vitvi02g04130\_t001 |  |  |  | \| \| |  |  |  |  |  |  |  |  |
| 1 | transcript:Vitvi02g04131\_t001 |  |  |  | \| \| |  |  |  |  |  |  |  |  |
| 2 | transcript:Vitvi02g04132\_t001 |  | transcript:Vitvi15g00684\_t001 |  | \| \| |  |  |  |  |  |  |  |  |
| 2 | transcript:Vitvi02g04133\_t001 |  | \| \| |  | \| \| |  |  |  |  |  |  |  |  |
| 2 | transcript:Vitvi02g04134\_t001 |  | \| \| |  | \| \| |  |  |  |  |  |  |  |  |
| 2 | transcript:Vitvi02g04135\_t001 |  | \| \| |  | \| \| |  |  |  |  |  |  |  |  |
| 2 | transcript:Vitvi02g04136\_t001 |  | \| \| |  | \| \| |  |  |  |  |  |  |  |  |
| 2 | transcript:Vitvi02g04137\_t001 |  | \| \| |  | \| \| |  |  |  |  |  |  |  |  |
| 2 | transcript:Vitvi02g04138\_t001 |  | \| \| |  | \| \| |  |  |  |  |  |  |  |  |
| 3 | transcript:Vitvi02g01823\_t001 |  | \| \| |  | \| \| |  | transcript:Vitvi16g00775\_t001 |  |  |  |  |  |  |
| 3 | transcript:Vitvi02g04139\_t001 |  | \| \| |  | \| \| |  | \| \| |  |  |  |  |  |  |
| 3 | transcript:Vitvi02g00496\_t001 |  | \| \| |  | \| \| |  | \| \| |  |  |  |  |  |  |
| 3 | transcript:Vitvi02g00495\_t001 |  | \| \| |  | \| \| |  | \| \| |  |  |  |  |  |  |
| 3 | transcript:Vitvi02g01427\_t001 |  | \| \| |  | transcript:Vitvi16g01818\_t001 |  | \| \| |  |  |  |  |  |  |
| 2 | transcript:Vitvi02g04140\_t001 |  | \| \| |  |  |  | \| \| |  |  |  |  |  |  |
| 2 | transcript:Vitvi02g04141\_t001 |  | \| \| |  |  |  | \| \| |  |  |  |  |  |  |
| 2 | transcript:Vitvi02g01429\_t003 |  | \| \| |  |  |  | \| \| |  |  |  |  |  |  |
| 2 | transcript:Vitvi02g04142\_t001 |  | \| \| |  |  |  | \| \| |  |  |  |  |  |  |
| 2 | transcript:Vitvi02g00498\_t001 |  | \| \| |  |  |  | \| \| |  |  |  |  |  |  |
| 2 | transcript:Vitvi02g00499\_t001 |  | transcript:Vitvi15g00698\_t001 |  |  |  | transcript:Vitvi16g01800\_t001 |  |  |  |  |  |  |
| 2 | transcript:Vitvi02g00500\_t001 |  | \| \| |  |  |  | transcript:Vitvi16g04295\_t001 |  |  |  |  |  |  |
| 2 | transcript:Vitvi02g00501\_t001 |  | \| \| |  |  |  | \| \| |  |  |  |  |  |  |
| 2 | transcript:Vitvi02g04143\_t001 |  | \| \| |  |  |  | \| \| |  |  |  |  |  |  |
| 2 | transcript:Vitvi02g04144\_t001 |  | \| \| |  |  |  | \| \| |  |  |  |  |  |  |
| 2 | transcript:Vitvi02g01431\_t001 |  | \| \| |  |  |  | \| \| |  |  |  |  |  |  |
| 2 | transcript:Vitvi02g00502\_t001 |  | \| \| |  |  |  | \| \| |  |  |  |  |  |  |
| 2 | transcript:Vitvi02g01852\_t001 |  | \| \| |  |  |  | \| \| |  |  |  |  |  |  |
| 2 | transcript:Vitvi02g00505\_t001 |  | \| \| |  |  |  | \| \| |  |  |  |  |  |  |
| 2 | transcript:Vitvi02g00507\_t001 |  | transcript:Vitvi15g00706\_t001 |  |  |  | \| \| |  |  |  |  |  |  |
| 2 | transcript:Vitvi02g00508\_t001 |  | \| \| |  |  |  | transcript:Vitvi16g00716\_t001 |  |  |  |  |  |  |
| 2 | transcript:Vitvi02g01432\_t001 |  | \| \| |  |  |  | \| \| |  |  |  |  |  |  |
| 2 | transcript:Vitvi02g00509\_t004 |  | \| \| |  |  |  | \| \| |  |  |  |  |  |  |
| 2 | transcript:Vitvi02g01433\_t001 |  | \| \| |  |  |  | \| \| |  |  |  |  |  |  |
| 2 | transcript:Vitvi02g00510\_t001 |  | transcript:Vitvi15g00708\_t002 |  |  |  | \| \| |  |  |  |  |  |  |
| 2 | transcript:Vitvi02g00511\_t001 |  | \| \| |  |  |  | transcript:Vitvi16g00715\_t001 |  |  |  |  |  |  |
| 2 | transcript:Vitvi02g00512\_t001 |  | \| \| |  |  |  | transcript:Vitvi16g00713\_t001 |  |  |  |  |  |  |
| 2 | transcript:Vitvi02g04145\_t001 |  | \| \| |  |  |  | \| \| |  |  |  |  |  |  |
| 2 | transcript:Vitvi02g04146\_t001 |  | \| \| |  |  |  | transcript:Vitvi16g00712\_t001 |  |  |  |  |  |  |
| 1 | transcript:Vitvi02g04147\_t001 |  | \| \| |  |  |  |  |  |  |  |  |  |  |
| 1 | transcript:Vitvi02g04148\_t001 |  | \| \| |  |  |  |  |  |  |  |  |  |  |
| 1 | transcript:Vitvi02g04149\_t001 |  | \| \| |  |  |  |  |  |  |  |  |  |  |
| 1 | transcript:Vitvi02g01697\_t001 |  | \| \| |  |  |  |  |  |  |  |  |  |  |
| 1 | transcript:Vitvi02g01696\_t001 |  | \| \| |  |  |  |  |  |  |  |  |  |  |
| 1 | transcript:Vitvi02g01695\_t001 |  | \| \| |  |  |  |  |  |  |  |  |  |  |
| 1 | transcript:Vitvi02g01693\_t001 |  | \| \| |  |  |  |  |  |  |  |  |  |  |
| 1 | transcript:Vitvi02g01692\_t001 |  | \| \| |  |  |  |  |  |  |  |  |  |  |
| 1 | transcript:Vitvi02g01691\_t001 |  | transcript:Vitvi15g00710\_t001 |  |  |  |  |  |  |  |  |  |  |
| 1 | transcript:Vitvi02g04150\_t001 |  | \| \| |  |  |  |  |  |  |  |  |  |  |
| 1 | transcript:Vitvi02g04151\_t001 |  | \| \| |  |  |  |  |  |  |  |  |  |  |
| 1 | transcript:Vitvi02g04152\_t001 |  | \| \| |  |  |  |  |  |  |  |  |  |  |
| 1 | transcript:Vitvi02g04153\_t001 |  | \| \| |  |  |  |  |  |  |  |  |  |  |
| 1 | transcript:Vitvi02g01689\_t001 |  | \| \| |  |  |  |  |  |  |  |  |  |  |
| 1 | transcript:Vitvi02g04154\_t001 |  | \| \| |  |  |  |  |  |  |  |  |  |  |
| 1 | transcript:Vitvi02g01687\_t001 |  | \| \| |  |  |  |  |  |  |  |  |  |  |
| 1 | transcript:Vitvi02g01685\_t001 |  | \| \| |  |  |  |  |  |  |  |  |  |  |
| 1 | transcript:Vitvi02g01683\_t001 |  | \| \| |  |  |  |  |  |  |  |  |  |  |
| 1 | transcript:Vitvi02g01682\_t002 |  | \| \| |  |  |  |  |  |  |  |  |  |  |
| 1 | transcript:Vitvi02g01847\_t001 |  | \| \| |  |  |  |  |  |  |  |  |  |  |
| 1 | transcript:Vitvi02g04155\_t001 |  | \| \| |  |  |  |  |  |  |  |  |  |  |
| 1 | transcript:Vitvi02g00526\_t001 |  | \| \| |  |  |  |  |  |  |  |  |  |  |
| 1 | transcript:Vitvi02g00527\_t001 |  | \| \| |  |  |  |  |  |  |  |  |  |  |
| 1 | transcript:Vitvi02g04156\_t002 |  | \| \| |  |  |  |  |  |  |  |  |  |  |
| 1 | transcript:Vitvi02g00529\_t001 |  | \| \| |  |  |  |  |  |  |  |  |  |  |
| 1 | transcript:Vitvi02g04157\_t001 |  | \| \| |  |  |  |  |  |  |  |  |  |  |
| 1 | transcript:Vitvi02g01438\_t001 |  | transcript:Vitvi15g00714\_t001 |  |  |  |  |  |  |  |  |  |  |
| 1 | transcript:Vitvi02g01439\_t001 |  | \| \| |  |  |  |  |  |  |  |  |  |  |
| 1 | transcript:Vitvi02g01440\_t001 |  | \| \| |  |  |  |  |  |  |  |  |  |  |
| 1 | transcript:Vitvi02g00531\_t001 |  | \| \| |  |  |  |  |  |  |  |  |  |  |
| 1 | transcript:Vitvi02g00532\_t001 |  | \| \| |  |  |  |  |  |  |  |  |  |  |
| 1 | transcript:Vitvi02g04158\_t001 |  | \| \| |  |  |  |  |  |  |  |  |  |  |
| 1 | transcript:Vitvi02g01441\_t002 |  | \| \| |  |  |  |  |  |  |  |  |  |  |
| 1 | transcript:Vitvi02g01442\_t001 |  | \| \| |  |  |  |  |  |  |  |  |  |  |
| 1 | transcript:Vitvi02g00533\_t001 |  | transcript:Vitvi15g00715\_t001 |  |  |  |  |  |  |  |  |  |  |
| 1 | transcript:Vitvi02g00534\_t001 |  | \| \| |  |  |  |  |  |  |  |  |  |  |
| 1 | transcript:Vitvi02g01443\_t001 |  | \| \| |  |  |  |  |  |  |  |  |  |  |
| 1 | transcript:Vitvi02g04159\_t001 |  | \| \| |  |  |  |  |  |  |  |  |  |  |
| 1 | transcript:Vitvi02g00535\_t001 |  | transcript:Vitvi15g00718\_t002 |  |  |  |  |  |  |  |  |  |  |
| 0 | transcript:Vitvi02g00536\_t002 |  |  |  |  |  |  |  |  |  |  |  |  |
| 0 | transcript:Vitvi02g04160\_t001 |  |  |  |  |  |  |  |  |  |  |  |  |
| 0 | transcript:Vitvi02g00538\_t001 |  |  |  |  |  |  |  |  |  |  |  |  |
| 0 | transcript:Vitvi02g04161\_t001 |  |  |  |  |  |  |  |  |  |  |  |  |
| 0 | transcript:Vitvi02g01445\_t001 |  |  |  |  |  |  |  |  |  |  |  |  |
| 0 | transcript:Vitvi02g00540\_t001 |  |  |  |  |  |  |  |  |  |  |  |  |
| 0 | transcript:Vitvi02g00541\_t001 |  |  |  |  |  |  |  |  |  |  |  |  |
| 0 | transcript:Vitvi02g00542\_t001 |  |  |  |  |  |  |  |  |  |  |  |  |
| 0 | transcript:Vitvi02g00543\_t001 |  |  |  |  |  |  |  |  |  |  |  |  |
| 0 | transcript:Vitvi02g01446\_t001 |  |  |  |  |  |  |  |  |  |  |  |  |
| 0 | transcript:Vitvi02g01447\_t001 |  |  |  |  |  |  |  |  |  |  |  |  |
| 0 | transcript:Vitvi02g00545\_t001 |  |  |  |  |  |  |  |  |  |  |  |  |
| 0 | transcript:Vitvi02g00546\_t001 |  |  |  |  |  |  |  |  |  |  |  |  |
| 0 | transcript:Vitvi02g00547\_t001 |  |  |  |  |  |  |  |  |  |  |  |  |
| 0 | transcript:Vitvi02g00548\_t001 |  |  |  |  |  |  |  |  |  |  |  |  |
| 0 | transcript:Vitvi02g00549\_t001 |  |  |  |  |  |  |  |  |  |  |  |  |
| 0 | transcript:Vitvi02g00551\_t001 |  |  |  |  |  |  |  |  |  |  |  |  |
| 0 | transcript:Vitvi02g04162\_t001 |  |  |  |  |  |  |  |  |  |  |  |  |
| 0 | transcript:Vitvi02g04163\_t001 |  |  |  |  |  |  |  |  |  |  |  |  |
| 0 | transcript:Vitvi02g04164\_t001 |  |  |  |  |  |  |  |  |  |  |  |  |
| 0 | transcript:Vitvi02g00553\_t001 |  |  |  |  |  |  |  |  |  |  |  |  |
| 0 | transcript:Vitvi02g00554\_t001 |  |  |  |  |  |  |  |  |  |  |  |  |
| 0 | transcript:Vitvi02g04165\_t001 |  |  |  |  |  |  |  |  |  |  |  |  |
| 0 | transcript:Vitvi02g00558\_t001 |  |  |  |  |  |  |  |  |  |  |  |  |
| 0 | transcript:Vitvi02g04166\_t001 |  |  |  |  |  |  |  |  |  |  |  |  |
| 0 | transcript:Vitvi02g04167\_t001 |  |  |  |  |  |  |  |  |  |  |  |  |
| 0 | transcript:Vitvi02g04168\_t001 |  |  |  |  |  |  |  |  |  |  |  |  |
| 0 | transcript:Vitvi02g01452\_t001 |  |  |  |  |  |  |  |  |  |  |  |  |
| 0 | transcript:Vitvi02g00560\_t001 |  |  |  |  |  |  |  |  |  |  |  |  |
| 0 | transcript:Vitvi02g04169\_t001 |  |  |  |  |  |  |  |  |  |  |  |  |
| 0 | transcript:Vitvi02g04170\_t001 |  |  |  |  |  |  |  |  |  |  |  |  |
| 0 | transcript:Vitvi02g00563\_t001 |  |  |  |  |  |  |  |  |  |  |  |  |
| 0 | transcript:Vitvi02g01706\_t006 |  |  |  |  |  |  |  |  |  |  |  |  |
| 0 | transcript:Vitvi02g00564\_t001 |  |  |  |  |  |  |  |  |  |  |  |  |
| 0 | transcript:Vitvi02g00565\_t001 |  |  |  |  |  |  |  |  |  |  |  |  |
| 0 | transcript:Vitvi02g00566\_t001 |  |  |  |  |  |  |  |  |  |  |  |  |
| 0 | transcript:Vitvi02g00567\_t001 |  |  |  |  |  |  |  |  |  |  |  |  |
| 0 | transcript:Vitvi02g00568\_t001 |  |  |  |  |  |  |  |  |  |  |  |  |
| 0 | transcript:Vitvi02g00569\_t001 |  |  |  |  |  |  |  |  |  |  |  |  |
| 0 | transcript:Vitvi02g00570\_t001 |  |  |  |  |  |  |  |  |  |  |  |  |
| 0 | transcript:Vitvi02g04171\_t001 |  |  |  |  |  |  |  |  |  |  |  |  |
| 0 | transcript:Vitvi02g00572\_t001 |  |  |  |  |  |  |  |  |  |  |  |  |
| 0 | transcript:Vitvi02g04172\_t001 |  |  |  |  |  |  |  |  |  |  |  |  |
| 0 | transcript:Vitvi02g00573\_t001 |  |  |  |  |  |  |  |  |  |  |  |  |
| 0 | transcript:Vitvi02g00575\_t001 |  |  |  |  |  |  |  |  |  |  |  |  |
| 0 | transcript:Vitvi02g04173\_t001 |  |  |  |  |  |  |  |  |  |  |  |  |
| 0 | transcript:Vitvi02g01712\_t001 |  |  |  |  |  |  |  |  |  |  |  |  |
| 0 | transcript:Vitvi02g01713\_t001 |  |  |  |  |  |  |  |  |  |  |  |  |
| 0 | transcript:Vitvi02g01714\_t001 |  |  |  |  |  |  |  |  |  |  |  |  |
| 0 | transcript:Vitvi02g01716\_t001 |  |  |  |  |  |  |  |  |  |  |  |  |
| 0 | transcript:Vitvi02g01717\_t001 |  |  |  |  |  |  |  |  |  |  |  |  |
| 0 | transcript:Vitvi02g04174\_t001 |  |  |  |  |  |  |  |  |  |  |  |  |
| 0 | transcript:Vitvi02g04175\_t001 |  |  |  |  |  |  |  |  |  |  |  |  |
| 0 | transcript:Vitvi02g01718\_t001 |  |  |  |  |  |  |  |  |  |  |  |  |
| 0 | transcript:Vitvi02g04176\_t001 |  |  |  |  |  |  |  |  |  |  |  |  |
| 0 | transcript:Vitvi02g01720\_t001 |  |  |  |  |  |  |  |  |  |  |  |  |
| 0 | transcript:Vitvi02g01721\_t001 |  |  |  |  |  |  |  |  |  |  |  |  |
| 0 | transcript:Vitvi02g04177\_t001 |  |  |  |  |  |  |  |  |  |  |  |  |
| 0 | transcript:Vitvi02g01723\_t001 |  |  |  |  |  |  |  |  |  |  |  |  |
| 0 | transcript:Vitvi02g01726\_t001 |  |  |  |  |  |  |  |  |  |  |  |  |
| 0 | transcript:Vitvi02g01727\_t001 |  |  |  |  |  |  |  |  |  |  |  |  |
| 0 | transcript:Vitvi02g01728\_t001 |  |  |  |  |  |  |  |  |  |  |  |  |
| 0 | transcript:Vitvi02g04178\_t001 |  |  |  |  |  |  |  |  |  |  |  |  |
| 0 | transcript:Vitvi02g04179\_t001 |  |  |  |  |  |  |  |  |  |  |  |  |
| 0 | transcript:Vitvi02g04180\_t001 |  |  |  |  |  |  |  |  |  |  |  |  |
| 0 | transcript:Vitvi02g04181\_t001 |  |  |  |  |  |  |  |  |  |  |  |  |
| 0 | transcript:Vitvi02g00590\_t001 |  |  |  |  |  |  |  |  |  |  |  |  |
| 0 | transcript:Vitvi02g00593\_t001 |  |  |  |  |  |  |  |  |  |  |  |  |
| 0 | transcript:Vitvi02g04182\_t001 |  |  |  |  |  |  |  |  |  |  |  |  |
| 0 | transcript:Vitvi02g00595\_t001 |  |  |  |  |  |  |  |  |  |  |  |  |
| 0 | transcript:Vitvi02g04183\_t001 |  |  |  |  |  |  |  |  |  |  |  |  |
| 0 | transcript:Vitvi02g00597\_t001 |  |  |  |  |  |  |  |  |  |  |  |  |
| 0 | transcript:Vitvi02g00598\_t003 |  |  |  |  |  |  |  |  |  |  |  |  |
| 0 | transcript:Vitvi02g00599\_t001 |  |  |  |  |  |  |  |  |  |  |  |  |
| 0 | transcript:Vitvi02g00600\_t001 |  |  |  |  |  |  |  |  |  |  |  |  |
| 0 | transcript:Vitvi02g00603\_t001 |  |  |  |  |  |  |  |  |  |  |  |  |
| 0 | transcript:Vitvi02g00604\_t001 |  |  |  |  |  |  |  |  |  |  |  |  |
| 0 | transcript:Vitvi02g01470\_t001 |  |  |  |  |  |  |  |  |  |  |  |  |
| 0 | transcript:Vitvi02g00605\_t001 |  |  |  |  |  |  |  |  |  |  |  |  |
| 0 | transcript:Vitvi02g00606\_t001 |  |  |  |  |  |  |  |  |  |  |  |  |
| 0 | transcript:Vitvi02g00607\_t001 |  |  |  |  |  |  |  |  |  |  |  |  |
| 0 | transcript:Vitvi02g00614\_t001 |  |  |  |  |  |  |  |  |  |  |  |  |
| 0 | transcript:Vitvi02g01471\_t001 |  |  |  |  |  |  |  |  |  |  |  |  |
| 0 | transcript:Vitvi02g00615\_t001 |  |  |  |  |  |  |  |  |  |  |  |  |
| 0 | transcript:Vitvi02g00617\_t001 |  |  |  |  |  |  |  |  |  |  |  |  |
| 0 | transcript:Vitvi02g00618\_t001 |  |  |  |  |  |  |  |  |  |  |  |  |
| 0 | transcript:Vitvi02g00619\_t001 |  |  |  |  |  |  |  |  |  |  |  |  |
| 0 | transcript:Vitvi02g00620\_t001 |  |  |  |  |  |  |  |  |  |  |  |  |
| 0 | transcript:Vitvi02g04184\_t001 |  |  |  |  |  |  |  |  |  |  |  |  |
| 0 | transcript:Vitvi02g01472\_t001 |  |  |  |  |  |  |  |  |  |  |  |  |
| 0 | transcript:Vitvi02g01473\_t001 |  |  |  |  |  |  |  |  |  |  |  |  |
| 0 | transcript:Vitvi02g01474\_t001 |  |  |  |  |  |  |  |  |  |  |  |  |
| 0 | transcript:Vitvi02g01479\_t001 |  |  |  |  |  |  |  |  |  |  |  |  |
| 0 | transcript:Vitvi02g00626\_t001 |  |  |  |  |  |  |  |  |  |  |  |  |
| 0 | transcript:Vitvi02g04185\_t001 |  |  |  |  |  |  |  |  |  |  |  |  |
| 0 | transcript:Vitvi02g01480\_t001 |  |  |  |  |  |  |  |  |  |  |  |  |
| 0 | transcript:Vitvi02g00627\_t001 |  |  |  |  |  |  |  |  |  |  |  |  |
| 0 | transcript:Vitvi02g00628\_t001 |  |  |  |  |  |  |  |  |  |  |  |  |
| 0 | transcript:Vitvi02g01481\_t001 |  |  |  |  |  |  |  |  |  |  |  |  |
| 0 | transcript:Vitvi02g00629\_t001 |  |  |  |  |  |  |  |  |  |  |  |  |
| 0 | transcript:Vitvi02g01482\_t001 |  |  |  |  |  |  |  |  |  |  |  |  |
| 0 | transcript:Vitvi02g00630\_t001 |  |  |  |  |  |  |  |  |  |  |  |  |
| 0 | transcript:Vitvi02g00633\_t001 |  |  |  |  |  |  |  |  |  |  |  |  |
| 0 | transcript:Vitvi02g00634\_t001 |  |  |  |  |  |  |  |  |  |  |  |  |
| 0 | transcript:Vitvi02g01483\_t001 |  |  |  |  |  |  |  |  |  |  |  |  |
| 0 | transcript:Vitvi02g00635\_t001 |  |  |  |  |  |  |  |  |  |  |  |  |
| 0 | transcript:Vitvi02g04186\_t001 |  |  |  |  |  |  |  |  |  |  |  |  |
| 0 | transcript:Vitvi02g00636\_t002 |  |  |  |  |  |  |  |  |  |  |  |  |
| 0 | transcript:Vitvi02g04187\_t001 |  |  |  |  |  |  |  |  |  |  |  |  |
| 0 | transcript:Vitvi02g00640\_t001 |  |  |  |  |  |  |  |  |  |  |  |  |
| 0 | transcript:Vitvi02g00641\_t001 |  |  |  |  |  |  |  |  |  |  |  |  |
| 0 | transcript:Vitvi02g00642\_t001 |  |  |  |  |  |  |  |  |  |  |  |  |
| 0 | transcript:Vitvi02g00645\_t001 |  |  |  |  |  |  |  |  |  |  |  |  |
| 0 | transcript:Vitvi02g01484\_t001 |  |  |  |  |  |  |  |  |  |  |  |  |
| 0 | transcript:Vitvi02g01485\_t001 |  |  |  |  |  |  |  |  |  |  |  |  |
| 0 | transcript:Vitvi02g01486\_t001 |  |  |  |  |  |  |  |  |  |  |  |  |
| 0 | transcript:Vitvi02g04188\_t001 |  |  |  |  |  |  |  |  |  |  |  |  |
| 0 | transcript:Vitvi02g04189\_t001 |  |  |  |  |  |  |  |  |  |  |  |  |
| 0 | transcript:Vitvi02g01488\_t001 |  |  |  |  |  |  |  |  |  |  |  |  |
| 0 | transcript:Vitvi02g04190\_t001 |  |  |  |  |  |  |  |  |  |  |  |  |
| 0 | transcript:Vitvi02g04191\_t001 |  |  |  |  |  |  |  |  |  |  |  |  |
| 0 | transcript:Vitvi02g01492\_t001 |  |  |  |  |  |  |  |  |  |  |  |  |
| 0 | transcript:Vitvi02g00648\_t001 |  |  |  |  |  |  |  |  |  |  |  |  |
| 0 | transcript:Vitvi02g00649\_t001 |  |  |  |  |  |  |  |  |  |  |  |  |
| 0 | transcript:Vitvi02g00650\_t003 |  |  |  |  |  |  |  |  |  |  |  |  |
| 0 | transcript:Vitvi02g04192\_t001 |  |  |  |  |  |  |  |  |  |  |  |  |
| 0 | transcript:Vitvi02g00653\_t001 |  |  |  |  |  |  |  |  |  |  |  |  |
| 0 | transcript:Vitvi02g00654\_t001 |  |  |  |  |  |  |  |  |  |  |  |  |
| 0 | transcript:Vitvi02g00655\_t001 |  |  |  |  |  |  |  |  |  |  |  |  |
| 0 | transcript:Vitvi02g04193\_t001 |  |  |  |  |  |  |  |  |  |  |  |  |
| 0 | transcript:Vitvi02g00656\_t001 |  |  |  |  |  |  |  |  |  |  |  |  |
| 0 | transcript:Vitvi02g00658\_t001 |  |  |  |  |  |  |  |  |  |  |  |  |
| 0 | transcript:Vitvi02g00659\_t001 |  |  |  |  |  |  |  |  |  |  |  |  |
| 0 | transcript:Vitvi02g00660\_t001 |  |  |  |  |  |  |  |  |  |  |  |  |
| 0 | transcript:Vitvi02g00661\_t001 |  |  |  |  |  |  |  |  |  |  |  |  |
| 0 | transcript:Vitvi02g00662\_t001 |  |  |  |  |  |  |  |  |  |  |  |  |
| 0 | transcript:Vitvi02g00663\_t001 |  |  |  |  |  |  |  |  |  |  |  |  |
| 0 | transcript:Vitvi02g01498\_t001 |  |  |  |  |  |  |  |  |  |  |  |  |
| 0 | transcript:Vitvi02g04194\_t001 |  |  |  |  |  |  |  |  |  |  |  |  |
| 0 | transcript:Vitvi02g00664\_t001 |  |  |  |  |  |  |  |  |  |  |  |  |
| 0 | transcript:Vitvi02g01499\_t001 |  |  |  |  |  |  |  |  |  |  |  |  |
| 0 | transcript:Vitvi02g04195\_t001 |  |  |  |  |  |  |  |  |  |  |  |  |
| 0 | transcript:Vitvi02g00665\_t001 |  |  |  |  |  |  |  |  |  |  |  |  |
| 0 | transcript:Vitvi02g00666\_t003 |  |  |  |  |  |  |  |  |  |  |  |  |
| 0 | transcript:Vitvi02g00667\_t001 |  |  |  |  |  |  |  |  |  |  |  |  |
| 0 | transcript:Vitvi02g04196\_t001 |  |  |  |  |  |  |  |  |  |  |  |  |
| 0 | transcript:Vitvi02g00669\_t001 |  |  |  |  |  |  |  |  |  |  |  |  |
| 0 | transcript:Vitvi02g00670\_t001 |  |  |  |  |  |  |  |  |  |  |  |  |
| 0 | transcript:Vitvi02g04197\_t001 |  |  |  |  |  |  |  |  |  |  |  |  |
| 0 | transcript:Vitvi02g04198\_t001 |  |  |  |  |  |  |  |  |  |  |  |  |
| 0 | transcript:Vitvi02g04199\_t001 |  |  |  |  |  |  |  |  |  |  |  |  |
| 0 | transcript:Vitvi02g00671\_t001 |  |  |  |  |  |  |  |  |  |  |  |  |
| 0 | transcript:Vitvi02g00672\_t001 |  |  |  |  |  |  |  |  |  |  |  |  |
| 0 | transcript:Vitvi02g00673\_t001 |  |  |  |  |  |  |  |  |  |  |  |  |
| 0 | transcript:Vitvi02g00674\_t001 |  |  |  |  |  |  |  |  |  |  |  |  |
| 0 | transcript:Vitvi02g04200\_t001 |  |  |  |  |  |  |  |  |  |  |  |  |
| 0 | transcript:Vitvi02g04201\_t001 |  |  |  |  |  |  |  |  |  |  |  |  |
| 0 | transcript:Vitvi02g00677\_t001 |  |  |  |  |  |  |  |  |  |  |  |  |
| 0 | transcript:Vitvi02g00678\_t001 |  |  |  |  |  |  |  |  |  |  |  |  |
| 0 | transcript:Vitvi02g00679\_t002 |  |  |  |  |  |  |  |  |  |  |  |  |
| 0 | transcript:Vitvi02g00680\_t001 |  |  |  |  |  |  |  |  |  |  |  |  |
| 0 | transcript:Vitvi02g00682\_t001 |  |  |  |  |  |  |  |  |  |  |  |  |
| 0 | transcript:Vitvi02g00683\_t001 |  |  |  |  |  |  |  |  |  |  |  |  |
| 0 | transcript:Vitvi02g00684\_t001 |  |  |  |  |  |  |  |  |  |  |  |  |
| 0 | transcript:Vitvi02g00685\_t001 |  |  |  |  |  |  |  |  |  |  |  |  |
| 0 | transcript:Vitvi02g00686\_t001 |  |  |  |  |  |  |  |  |  |  |  |  |
| 0 | transcript:Vitvi02g00688\_t001 |  |  |  |  |  |  |  |  |  |  |  |  |
| 0 | transcript:Vitvi02g00689\_t001 |  |  |  |  |  |  |  |  |  |  |  |  |
| 1 | transcript:Vitvi02g00691\_t003 |  | transcript:Vitvi16g00076\_t001 |  |  |  |  |  |  |  |  |  |  |
| 1 | transcript:Vitvi02g00693\_t001 |  | \| \| |  |  |  |  |  |  |  |  |  |  |
| 1 | transcript:Vitvi02g00694\_t001 |  | \| \| |  |  |  |  |  |  |  |  |  |  |
| 1 | transcript:Vitvi02g00695\_t001 |  | \| \| |  |  |  |  |  |  |  |  |  |  |
| 1 | transcript:Vitvi02g00696\_t001 |  | \| \| |  |  |  |  |  |  |  |  |  |  |
| 1 | transcript:Vitvi02g01504\_t001 |  | \| \| |  |  |  |  |  |  |  |  |  |  |
| 1 | transcript:Vitvi02g01505\_t001 |  | \| \| |  |  |  |  |  |  |  |  |  |  |
| 1 | transcript:Vitvi02g00698\_t001 |  | \| \| |  |  |  |  |  |  |  |  |  |  |
| 1 | transcript:Vitvi02g00699\_t001 |  | \| \| |  |  |  |  |  |  |  |  |  |  |
| 1 | transcript:Vitvi02g00700\_t001 |  | \| \| |  |  |  |  |  |  |  |  |  |  |
| 1 | transcript:Vitvi02g01506\_t001 |  | \| \| |  |  |  |  |  |  |  |  |  |  |
| 1 | transcript:Vitvi02g04202\_t001 |  | \| \| |  |  |  |  |  |  |  |  |  |  |
| 1 | transcript:Vitvi02g00701\_t001 |  | \| \| |  |  |  |  |  |  |  |  |  |  |
| 1 | transcript:Vitvi02g01507\_t001 |  | \| \| |  |  |  |  |  |  |  |  |  |  |
| 1 | transcript:Vitvi02g00702\_t001 |  | \| \| |  |  |  |  |  |  |  |  |  |  |
| 1 | transcript:Vitvi02g00703\_t001 |  | \| \| |  |  |  |  |  |  |  |  |  |  |
| 1 | transcript:Vitvi02g00704\_t001 |  | transcript:Vitvi16g00083\_t001 |  |  |  |  |  |  |  |  |  |  |
| 1 | transcript:Vitvi02g00705\_t001 |  | \| \| |  |  |  |  |  |  |  |  |  |  |
| 1 | transcript:Vitvi02g00706\_t001 |  | \| \| |  |  |  |  |  |  |  |  |  |  |
| 1 | transcript:Vitvi02g00708\_t001 |  | \| \| |  |  |  |  |  |  |  |  |  |  |
| 1 | transcript:Vitvi02g00709\_t001 |  | \| \| |  |  |  |  |  |  |  |  |  |  |
| 1 | transcript:Vitvi02g00710\_t001 |  | \| \| |  |  |  |  |  |  |  |  |  |  |
| 1 | transcript:Vitvi02g04203\_t001 |  | \| \| |  |  |  |  |  |  |  |  |  |  |
| 1 | transcript:Vitvi02g04204\_t001 |  | \| \| |  |  |  |  |  |  |  |  |  |  |
| 1 | transcript:Vitvi02g00711\_t001 |  | \| \| |  |  |  |  |  |  |  |  |  |  |
| 1 | transcript:Vitvi02g04205\_t001 |  | \| \| |  |  |  |  |  |  |  |  |  |  |
| 1 | transcript:Vitvi02g04206\_t001 |  | \| \| |  |  |  |  |  |  |  |  |  |  |
| 1 | transcript:Vitvi02g00713\_t001 |  | \| \| |  |  |  |  |  |  |  |  |  |  |
| 1 | transcript:Vitvi02g00714\_t001 |  | \| \| |  |  |  |  |  |  |  |  |  |  |
| 1 | transcript:Vitvi02g00715\_t001 |  | transcript:Vitvi16g00084\_t001 |  |  |  |  |  |  |  |  |  |  |
| 1 | transcript:Vitvi02g00717\_t002 |  | transcript:Vitvi16g00095\_t001 |  |  |  |  |  |  |  |  |  |  |
| 1 | transcript:Vitvi02g00718\_t001 |  | \| \| |  |  |  |  |  |  |  |  |  |  |
| 1 | transcript:Vitvi02g04207\_t001 |  | \| \| |  |  |  |  |  |  |  |  |  |  |
| 1 | transcript:Vitvi02g00719\_t001 |  | \| \| |  |  |  |  |  |  |  |  |  |  |
| 1 | transcript:Vitvi02g00721\_t001 |  | \| \| |  |  |  |  |  |  |  |  |  |  |
| 1 | transcript:Vitvi02g00722\_t001 |  | \| \| |  |  |  |  |  |  |  |  |  |  |
| 1 | transcript:Vitvi02g00723\_t001 |  | \| \| |  |  |  |  |  |  |  |  |  |  |
| 1 | transcript:Vitvi02g00724\_t001 |  | transcript:Vitvi16g00097\_t001 |  |  |  |  |  |  |  |  |  |  |
| 1 | transcript:Vitvi02g00725\_t001 |  | transcript:Vitvi16g00098\_t001 |  |  |  |  |  |  |  |  |  |  |
| 0 | transcript:Vitvi02g00726\_t001 |  |  |  |  |  |  |  |  |  |  |  |  |
| 0 | transcript:Vitvi02g00728\_t001 |  |  |  |  |  |  |  |  |  |  |  |  |
| 0 | transcript:Vitvi02g00729\_t003 |  |  |  |  |  |  |  |  |  |  |  |  |
| 0 | transcript:Vitvi02g04208\_t001 |  |  |  |  |  |  |  |  |  |  |  |  |
| 0 | transcript:Vitvi02g04209\_t001 |  |  |  |  |  |  |  |  |  |  |  |  |
| 0 | transcript:Vitvi02g00732\_t001 |  |  |  |  |  |  |  |  |  |  |  |  |
| 0 | transcript:Vitvi02g00733\_t001 |  |  |  |  |  |  |  |  |  |  |  |  |
| 0 | transcript:Vitvi02g04210\_t001 |  |  |  |  |  |  |  |  |  |  |  |  |
| 0 | transcript:Vitvi02g00734\_t001 |  |  |  |  |  |  |  |  |  |  |  |  |
| 0 | transcript:Vitvi02g00735\_t001 |  |  |  |  |  |  |  |  |  |  |  |  |
| 0 | transcript:Vitvi02g04211\_t001 |  |  |  |  |  |  |  |  |  |  |  |  |
| 0 | transcript:Vitvi02g00737\_t001 |  |  |  |  |  |  |  |  |  |  |  |  |
| 0 | transcript:Vitvi02g00738\_t001 |  |  |  |  |  |  |  |  |  |  |  |  |
| 0 | transcript:Vitvi02g00739\_t001 |  |  |  |  |  |  |  |  |  |  |  |  |
| 0 | transcript:Vitvi02g04212\_t001 |  |  |  |  |  |  |  |  |  |  |  |  |
| 0 | transcript:Vitvi02g00741\_t001 |  |  |  |  |  |  |  |  |  |  |  |  |
| 0 | transcript:Vitvi02g00744\_t001 |  |  |  |  |  |  |  |  |  |  |  |  |
| 0 | transcript:Vitvi02g00746\_t001 |  |  |  |  |  |  |  |  |  |  |  |  |
| 0 | transcript:Vitvi02g00747\_t001 |  |  |  |  |  |  |  |  |  |  |  |  |
| 0 | transcript:Vitvi02g00749\_t001 |  |  |  |  |  |  |  |  |  |  |  |  |
| 0 | transcript:Vitvi02g01513\_t001 |  |  |  |  |  |  |  |  |  |  |  |  |
| 0 | transcript:Vitvi02g01514\_t002 |  |  |  |  |  |  |  |  |  |  |  |  |
| 0 | transcript:Vitvi02g04213\_t001 |  |  |  |  |  |  |  |  |  |  |  |  |
| 0 | transcript:Vitvi02g00750\_t001 |  |  |  |  |  |  |  |  |  |  |  |  |
| 0 | transcript:Vitvi02g01515\_t001 |  |  |  |  |  |  |  |  |  |  |  |  |
| 0 | transcript:Vitvi02g00751\_t001 |  |  |  |  |  |  |  |  |  |  |  |  |
| 0 | transcript:Vitvi02g00752\_t001 |  |  |  |  |  |  |  |  |  |  |  |  |
| 0 | transcript:Vitvi02g00757\_t002 |  |  |  |  |  |  |  |  |  |  |  |  |
| 0 | transcript:Vitvi02g00758\_t001 |  |  |  |  |  |  |  |  |  |  |  |  |
| 0 | transcript:Vitvi02g00759\_t001 |  |  |  |  |  |  |  |  |  |  |  |  |
| 0 | transcript:Vitvi02g00762\_t001 |  |  |  |  |  |  |  |  |  |  |  |  |
| 0 | transcript:Vitvi02g04214\_t001 |  |  |  |  |  |  |  |  |  |  |  |  |
| 0 | transcript:Vitvi02g00763\_t001 |  |  |  |  |  |  |  |  |  |  |  |  |
| 0 | transcript:Vitvi02g00768\_t001 |  |  |  |  |  |  |  |  |  |  |  |  |
| 0 | transcript:Vitvi02g00771\_t001 |  |  |  |  |  |  |  |  |  |  |  |  |
| 0 | transcript:Vitvi02g00772\_t002 |  |  |  |  |  |  |  |  |  |  |  |  |
| 0 | transcript:Vitvi02g00773\_t001 |  |  |  |  |  |  |  |  |  |  |  |  |
| 0 | transcript:Vitvi02g00774\_t001 |  |  |  |  |  |  |  |  |  |  |  |  |
| 0 | transcript:Vitvi02g00775\_t001 |  |  |  |  |  |  |  |  |  |  |  |  |
| 0 | transcript:Vitvi02g00777\_t001 |  |  |  |  |  |  |  |  |  |  |  |  |
| 0 | transcript:Vitvi02g00780\_t001 |  |  |  |  |  |  |  |  |  |  |  |  |
| 0 | transcript:Vitvi02g04216\_t001 |  |  |  |  |  |  |  |  |  |  |  |  |
| 0 | transcript:Vitvi02g00783\_t001 |  |  |  |  |  |  |  |  |  |  |  |  |
| 0 | transcript:Vitvi02g00784\_t001 |  |  |  |  |  |  |  |  |  |  |  |  |
| 0 | transcript:Vitvi02g00785\_t001 |  |  |  |  |  |  |  |  |  |  |  |  |
| 0 | transcript:Vitvi02g00787\_t001 |  |  |  |  |  |  |  |  |  |  |  |  |
| 0 | transcript:Vitvi02g00790\_t001 |  |  |  |  |  |  |  |  |  |  |  |  |
| 0 | transcript:Vitvi02g00792\_t001 |  |  |  |  |  |  |  |  |  |  |  |  |
| 0 | transcript:Vitvi02g00795\_t001 |  |  |  |  |  |  |  |  |  |  |  |  |
| 0 | transcript:Vitvi02g00796\_t003 |  |  |  |  |  |  |  |  |  |  |  |  |
| 0 | transcript:Vitvi02g00798\_t001 |  |  |  |  |  |  |  |  |  |  |  |  |
| 0 | transcript:Vitvi02g00802\_t001 |  |  |  |  |  |  |  |  |  |  |  |  |
| 0 | transcript:Vitvi02g04217\_t001 |  |  |  |  |  |  |  |  |  |  |  |  |
| 0 | transcript:Vitvi02g04218\_t001 |  |  |  |  |  |  |  |  |  |  |  |  |
| 0 | transcript:Vitvi02g00805\_t001 |  |  |  |  |  |  |  |  |  |  |  |  |
| 0 | transcript:Vitvi02g04219\_t001 |  |  |  |  |  |  |  |  |  |  |  |  |
| 0 | transcript:Vitvi02g04220\_t001 |  |  |  |  |  |  |  |  |  |  |  |  |
| 0 | transcript:Vitvi02g00808\_t001 |  |  |  |  |  |  |  |  |  |  |  |  |
| 0 | transcript:Vitvi02g00809\_t001 |  |  |  |  |  |  |  |  |  |  |  |  |
| 0 | transcript:Vitvi02g04221\_t001 |  |  |  |  |  |  |  |  |  |  |  |  |
| 0 | transcript:Vitvi02g01526\_t001 |  |  |  |  |  |  |  |  |  |  |  |  |
| 0 | transcript:Vitvi02g04222\_t001 |  |  |  |  |  |  |  |  |  |  |  |  |
| 0 | transcript:Vitvi02g04223\_t001 |  |  |  |  |  |  |  |  |  |  |  |  |
| 0 | transcript:Vitvi02g04224\_t001 |  |  |  |  |  |  |  |  |  |  |  |  |
| 0 | transcript:Vitvi02g04225\_t001 |  |  |  |  |  |  |  |  |  |  |  |  |
| 0 | transcript:Vitvi02g00818\_t001 |  |  |  |  |  |  |  |  |  |  |  |  |
| 0 | transcript:Vitvi02g04226\_t001 |  |  |  |  |  |  |  |  |  |  |  |  |
| 0 | transcript:Vitvi02g04227\_t001 |  |  |  |  |  |  |  |  |  |  |  |  |
| 0 | transcript:Vitvi02g04228\_t001 |  |  |  |  |  |  |  |  |  |  |  |  |
| 0 | transcript:Vitvi02g04229\_t001 |  |  |  |  |  |  |  |  |  |  |  |  |
| 0 | transcript:Vitvi02g04230\_t001 |  |  |  |  |  |  |  |  |  |  |  |  |
| 0 | transcript:Vitvi02g04231\_t001 |  |  |  |  |  |  |  |  |  |  |  |  |
| 0 | transcript:Vitvi02g04232\_t001 |  |  |  |  |  |  |  |  |  |  |  |  |
| 0 | transcript:Vitvi02g04233\_t001 |  |  |  |  |  |  |  |  |  |  |  |  |
| 0 | transcript:Vitvi02g01528\_t001 |  |  |  |  |  |  |  |  |  |  |  |  |
| 0 | transcript:Vitvi02g00828\_t002 |  |  |  |  |  |  |  |  |  |  |  |  |
| 0 | transcript:Vitvi02g00831\_t001 |  |  |  |  |  |  |  |  |  |  |  |  |
| 0 | transcript:Vitvi02g04234\_t001 |  |  |  |  |  |  |  |  |  |  |  |  |
| 0 | transcript:Vitvi02g01529\_t001 |  |  |  |  |  |  |  |  |  |  |  |  |
| 0 | transcript:Vitvi02g04235\_t001 |  |  |  |  |  |  |  |  |  |  |  |  |
| 0 | transcript:Vitvi02g04237\_t001 |  |  |  |  |  |  |  |  |  |  |  |  |
| 0 | transcript:Vitvi02g04238\_t001 |  |  |  |  |  |  |  |  |  |  |  |  |
| 0 | transcript:Vitvi02g00836\_t001 |  |  |  |  |  |  |  |  |  |  |  |  |
| 0 | transcript:Vitvi02g01532\_t001 |  |  |  |  |  |  |  |  |  |  |  |  |
| 0 | transcript:Vitvi02g01533\_t001 |  |  |  |  |  |  |  |  |  |  |  |  |
| 0 | transcript:Vitvi02g00839\_t001 |  |  |  |  |  |  |  |  |  |  |  |  |
| 0 | transcript:Vitvi02g00840\_t001 |  |  |  |  |  |  |  |  |  |  |  |  |
| 0 | transcript:Vitvi02g04239\_t001 |  |  |  |  |  |  |  |  |  |  |  |  |
| 0 | transcript:Vitvi02g04240\_t001 |  |  |  |  |  |  |  |  |  |  |  |  |
| 0 | transcript:Vitvi02g00842\_t001 |  |  |  |  |  |  |  |  |  |  |  |  |
| 0 | transcript:Vitvi02g00843\_t001 |  |  |  |  |  |  |  |  |  |  |  |  |
| 0 | transcript:Vitvi02g00847\_t001 |  |  |  |  |  |  |  |  |  |  |  |  |
| 0 | transcript:Vitvi02g00848\_t001 |  |  |  |  |  |  |  |  |  |  |  |  |
| 0 | transcript:Vitvi02g00851\_t001 |  |  |  |  |  |  |  |  |  |  |  |  |
| 0 | transcript:Vitvi02g04241\_t001 |  |  |  |  |  |  |  |  |  |  |  |  |
| 0 | transcript:Vitvi02g04242\_t001 |  |  |  |  |  |  |  |  |  |  |  |  |
| 0 | transcript:Vitvi02g00853\_t001 |  |  |  |  |  |  |  |  |  |  |  |  |
| 0 | transcript:Vitvi02g01536\_t001 |  |  |  |  |  |  |  |  |  |  |  |  |
| 0 | transcript:Vitvi02g00855\_t001 |  |  |  |  |  |  |  |  |  |  |  |  |
| 0 | transcript:Vitvi02g00857\_t001 |  |  |  |  |  |  |  |  |  |  |  |  |
| 0 | transcript:Vitvi02g00859\_t001 |  |  |  |  |  |  |  |  |  |  |  |  |
| 0 | transcript:Vitvi02g04243\_t001 |  |  |  |  |  |  |  |  |  |  |  |  |
| 1 | transcript:Vitvi02g01537\_t001 |  | transcript:Vitvi02g04284\_t001 |  |  |  |  |  |  |  |  |  |  |
| 1 | transcript:Vitvi02g04244\_t001 |  | \| \| |  |  |  |  |  |  |  |  |  |  |
| 1 | transcript:Vitvi02g00867\_t001 |  | \| \| |  |  |  |  |  |  |  |  |  |  |
| 1 | transcript:Vitvi02g04245\_t001 |  | \| \| |  |  |  |  |  |  |  |  |  |  |
| 1 | transcript:Vitvi02g00868\_t001 |  | \| \| |  |  |  |  |  |  |  |  |  |  |
| 1 | transcript:Vitvi02g04246\_t001 |  | \| \| |  |  |  |  |  |  |  |  |  |  |
| 1 | transcript:Vitvi02g04247\_t001 |  | \| \| |  |  |  |  |  |  |  |  |  |  |
| 1 | transcript:Vitvi02g04248\_t001 |  | \| \| |  |  |  |  |  |  |  |  |  |  |
| 1 | transcript:Vitvi02g04249\_t001 |  | \| \| |  |  |  |  |  |  |  |  |  |  |
| 1 | transcript:Vitvi02g04250\_t001 |  | \| \| |  |  |  |  |  |  |  |  |  |  |
| 1 | transcript:Vitvi02g04251\_t001 |  | \| \| |  |  |  |  |  |  |  |  |  |  |
| 3 | transcript:Vitvi02g00893\_t001 |  | \| \| |  | transcript:Vitvi09g04424\_t001 |  | transcript:Vitvi09g04409\_t001 |  |  |  |  |  |  |
| 4 | transcript:Vitvi02g01542\_t001 |  | \| \| |  | transcript:Vitvi09g01823\_t001 |  | transcript:Vitvi09g04410\_t001 |  | transcript:Vitvi00g04098\_t001 |  |  |  |  |
| 4 | transcript:Vitvi02g04252\_t001 |  | \| \| |  | transcript:Vitvi09g04423\_t001 |  | \| \| |  | \| \| |  |  |  |  |
| 4 | transcript:Vitvi02g01543\_t001 |  | \| \| |  | \| \| |  | \| \| |  | \| \| |  |  |  |  |
| 4 | transcript:Vitvi02g04253\_t001 |  | \| \| |  | \| \| |  | \| \| |  | \| \| |  |  |  |  |
| 4 | transcript:Vitvi02g04254\_t001 |  | \| \| |  | \| \| |  | \| \| |  | \| \| |  |  |  |  |
| 4 | transcript:Vitvi02g04255\_t001 |  | \| \| |  | \| \| |  | \| \| |  | \| \| |  |  |  |  |
| 4 | transcript:Vitvi02g04256\_t001 |  | transcript:Vitvi02g04280\_t001 |  | \| \| |  | \| \| |  | \| \| |  |  |  |  |
| 4 | transcript:Vitvi02g04257\_t001 |  | \| \| |  | \| \| |  | \| \| |  | \| \| |  |  |  |  |
| 4 | transcript:Vitvi02g00896\_t001 |  | transcript:Vitvi02g04268\_t001 |  | transcript:Vitvi09g01047\_t001 |  | transcript:Vitvi09g04411\_t001 |  | transcript:Vitvi00g04099\_t001 |  |  |  |  |
| 4 | transcript:Vitvi02g00897\_t001 |  | transcript:Vitvi02g04266\_t001 |  | \| \| |  | transcript:Vitvi09g04413\_t001 |  | transcript:Vitvi00g04101\_t001 |  |  |  |  |
| 4 | transcript:Vitvi02g04258\_t001 |  | transcript:Vitvi02g04265\_t001 |  | \| \| |  | \| \| |  | transcript:Vitvi00g04103\_t001 |  |  |  |  |
| 4 | transcript:Vitvi02g04259\_t001 |  | transcript:Vitvi02g04264\_t001 |  | \| \| |  | \| \| |  | transcript:Vitvi00g04104\_t001 |  |  |  |  |
| 4 | transcript:Vitvi02g04260\_t001 |  | transcript:Vitvi02g04263\_t001 |  | \| \| |  | transcript:Vitvi09g04417\_t001 |  | transcript:Vitvi00g04105\_t001 |  |  |  |  |
| 3 | transcript:Vitvi02g04261\_t001 |  |  |  | transcript:Vitvi09g04418\_t001 |  | transcript:Vitvi09g04418\_t001 |  | transcript:Vitvi00g04106\_t001 |  |  |  |  |
| 3 | transcript:Vitvi02g04262\_t001 |  |  |  | \| \| |  | \| \| |  | \| \| |  |  |  |  |
| 4 | transcript:Vitvi02g04263\_t001 |  | transcript:Vitvi02g04260\_t001 |  | \| \| |  | \| \| |  | \| \| |  |  |  |  |
| 4 | transcript:Vitvi02g04264\_t001 |  | transcript:Vitvi02g04259\_t001 |  | \| \| |  | \| \| |  | \| \| |  |  |  |  |
| 4 | transcript:Vitvi02g04265\_t001 |  | transcript:Vitvi02g04258\_t001 |  | \| \| |  | \| \| |  | \| \| |  |  |  |  |
| 4 | transcript:Vitvi02g04266\_t001 |  | transcript:Vitvi02g00897\_t001 |  | transcript:Vitvi09g04413\_t001 |  | transcript:Vitvi09g01047\_t001 |  | \| \| |  |  |  |  |
| 4 | transcript:Vitvi02g04267\_t001 |  | \| \| |  | transcript:Vitvi09g04412\_t001 |  | \| \| |  | \| \| |  |  |  |  |
| 4 | transcript:Vitvi02g04268\_t001 |  | transcript:Vitvi02g00896\_t001 |  | \| \| |  | transcript:Vitvi09g01823\_t001 |  | \| \| |  |  |  |  |
| 4 | transcript:Vitvi02g04269\_t001 |  | \| \| |  | \| \| |  | transcript:Vitvi09g04424\_t001 |  | \| \| |  |  |  |  |
| 3 | transcript:Vitvi02g04270\_t001 |  | \| \| |  | transcript:Vitvi09g04399\_t001 |  |  |  | \| \| |  |  |  |  |
| 2 | transcript:Vitvi02g04271\_t001 |  | \| \| |  |  |  |  |  | \| \| |  |  |  |  |
| 2 | transcript:Vitvi02g04272\_t001 |  | \| \| |  |  |  |  |  | \| \| |  |  |  |  |
| 2 | transcript:Vitvi02g04273\_t001 |  | \| \| |  |  |  |  |  | \| \| |  |  |  |  |
| 2 | transcript:Vitvi02g04274\_t001 |  | \| \| |  |  |  |  |  | \| \| |  |  |  |  |
| 2 | transcript:Vitvi02g01842\_t001 |  | \| \| |  |  |  |  |  | \| \| |  |  |  |  |
| 2 | transcript:Vitvi02g04276\_t001 |  | \| \| |  |  |  |  |  | \| \| |  |  |  |  |
| 2 | transcript:Vitvi02g00915\_t001 |  | \| \| |  |  |  |  |  | \| \| |  |  |  |  |
| 2 | transcript:Vitvi02g04277\_t001 |  | \| \| |  |  |  |  |  | \| \| |  |  |  |  |
| 2 | transcript:Vitvi02g00919\_t001 |  | \| \| |  |  |  |  |  | \| \| |  |  |  |  |
| 2 | transcript:Vitvi02g01556\_t001 |  | \| \| |  |  |  |  |  | \| \| |  |  |  |  |
| 2 | transcript:Vitvi02g04278\_t001 |  | \| \| |  |  |  |  |  | \| \| |  |  |  |  |
| 2 | transcript:Vitvi02g04279\_t001 |  | \| \| |  |  |  |  |  | \| \| |  |  |  |  |
| 2 | transcript:Vitvi02g00936\_t001 |  | \| \| |  |  |  |  |  | \| \| |  |  |  |  |
| 2 | transcript:Vitvi02g04280\_t001 |  | transcript:Vitvi02g04256\_t001 |  |  |  |  |  | transcript:Vitvi00g04131\_t001 |  |  |  |  |
| 1 | transcript:Vitvi02g04281\_t001 |  | \| \| |  |  |  |  |  |  |  |  |  |  |
| 1 | transcript:Vitvi02g00938\_t001 |  | \| \| |  |  |  |  |  |  |  |  |  |  |
| 1 | transcript:Vitvi02g04282\_t001 |  | \| \| |  |  |  |  |  |  |  |  |  |  |
| 1 | transcript:Vitvi02g04283\_t001 |  | \| \| |  |  |  |  |  |  |  |  |  |  |
| 1 | transcript:Vitvi02g01557\_t001 |  | \| \| |  |  |  |  |  |  |  |  |  |  |
| 1 | transcript:Vitvi02g04284\_t001 |  | transcript:Vitvi02g01537\_t001 |  |  |  |  |  |  |  |  |  |  |
| 0 | transcript:Vitvi02g00942\_t001 |  |  |  |  |  |  |  |  |  |  |  |  |
| 0 | transcript:Vitvi02g00945\_t001 |  |  |  |  |  |  |  |  |  |  |  |  |
| 0 | transcript:Vitvi02g01559\_t001 |  |  |  |  |  |  |  |  |  |  |  |  |
| 0 | transcript:Vitvi02g00950\_t001 |  |  |  |  |  |  |  |  |  |  |  |  |
| 0 | transcript:Vitvi02g00952\_t001 |  |  |  |  |  |  |  |  |  |  |  |  |
| 0 | transcript:Vitvi02g04285\_t001 |  |  |  |  |  |  |  |  |  |  |  |  |
| 0 | transcript:Vitvi02g00953\_t001 |  |  |  |  |  |  |  |  |  |  |  |  |
| 0 | transcript:Vitvi02g00954\_t001 |  |  |  |  |  |  |  |  |  |  |  |  |
| 0 | transcript:Vitvi02g01306\_t001 |  |  |  |  |  |  |  |  |  |  |  |  |
| 0 | transcript:Vitvi02g01303\_t001 |  |  |  |  |  |  |  |  |  |  |  |  |
| 0 | transcript:Vitvi02g04286\_t001 |  |  |  |  |  |  |  |  |  |  |  |  |
| 0 | transcript:Vitvi02g01567\_t001 |  |  |  |  |  |  |  |  |  |  |  |  |
| 0 | transcript:Vitvi02g01568\_t001 |  |  |  |  |  |  |  |  |  |  |  |  |
| 0 | transcript:Vitvi02g01569\_t001 |  |  |  |  |  |  |  |  |  |  |  |  |
| 0 | transcript:Vitvi02g04287\_t001 |  |  |  |  |  |  |  |  |  |  |  |  |
| 0 | transcript:Vitvi02g04288\_t001 |  |  |  |  |  |  |  |  |  |  |  |  |
| 0 | transcript:Vitvi02g04289\_t001 |  |  |  |  |  |  |  |  |  |  |  |  |
| 0 | transcript:Vitvi02g04290\_t001 |  |  |  |  |  |  |  |  |  |  |  |  |
| 0 | transcript:Vitvi02g01571\_t001 |  |  |  |  |  |  |  |  |  |  |  |  |
| 0 | transcript:Vitvi02g00963\_t001 |  |  |  |  |  |  |  |  |  |  |  |  |
| 0 | transcript:Vitvi02g04291\_t001 |  |  |  |  |  |  |  |  |  |  |  |  |
| 0 | transcript:Vitvi02g04292\_t001 |  |  |  |  |  |  |  |  |  |  |  |  |
| 0 | transcript:Vitvi02g04293\_t002 |  |  |  |  |  |  |  |  |  |  |  |  |
| 0 | transcript:Vitvi02g00972\_t001 |  |  |  |  |  |  |  |  |  |  |  |  |
| 0 | transcript:Vitvi02g00974\_t001 |  |  |  |  |  |  |  |  |  |  |  |  |
| 0 | transcript:Vitvi02g00975\_t001 |  |  |  |  |  |  |  |  |  |  |  |  |
| 0 | transcript:Vitvi02g00978\_t002 |  |  |  |  |  |  |  |  |  |  |  |  |
| 0 | transcript:Vitvi02g01575\_t001 |  |  |  |  |  |  |  |  |  |  |  |  |
| 0 | transcript:Vitvi02g04294\_t001 |  |  |  |  |  |  |  |  |  |  |  |  |
| 0 | transcript:Vitvi02g00982\_t002 |  |  |  |  |  |  |  |  |  |  |  |  |
| 0 | transcript:Vitvi02g00983\_t001 |  |  |  |  |  |  |  |  |  |  |  |  |
| 0 | transcript:Vitvi02g04295\_t001 |  |  |  |  |  |  |  |  |  |  |  |  |
| 0 | transcript:Vitvi02g00985\_t001 |  |  |  |  |  |  |  |  |  |  |  |  |
| 0 | transcript:Vitvi02g04296\_t001 |  |  |  |  |  |  |  |  |  |  |  |  |
| 0 | transcript:Vitvi02g01576\_t001 |  |  |  |  |  |  |  |  |  |  |  |  |
| 0 | transcript:Vitvi02g01577\_t001 |  |  |  |  |  |  |  |  |  |  |  |  |
| 0 | transcript:Vitvi02g04297\_t001 |  |  |  |  |  |  |  |  |  |  |  |  |
| 0 | transcript:Vitvi02g00991\_t001 |  |  |  |  |  |  |  |  |  |  |  |  |
| 0 | transcript:Vitvi02g00992\_t001 |  |  |  |  |  |  |  |  |  |  |  |  |
| 0 | transcript:Vitvi02g00994\_t001 |  |  |  |  |  |  |  |  |  |  |  |  |
| 0 | transcript:Vitvi02g00995\_t001 |  |  |  |  |  |  |  |  |  |  |  |  |
| 0 | transcript:Vitvi02g04298\_t001 |  |  |  |  |  |  |  |  |  |  |  |  |
| 0 | transcript:Vitvi02g04299\_t001 |  |  |  |  |  |  |  |  |  |  |  |  |
| 0 | transcript:Vitvi02g04300\_t001 |  |  |  |  |  |  |  |  |  |  |  |  |
| 0 | transcript:Vitvi02g04301\_t001 |  |  |  |  |  |  |  |  |  |  |  |  |
| 0 | transcript:Vitvi02g00998\_t001 |  |  |  |  |  |  |  |  |  |  |  |  |
| 0 | transcript:Vitvi02g00999\_t001 |  |  |  |  |  |  |  |  |  |  |  |  |
| 0 | transcript:Vitvi02g01001\_t001 |  |  |  |  |  |  |  |  |  |  |  |  |
| 0 | transcript:Vitvi02g01003\_t001 |  |  |  |  |  |  |  |  |  |  |  |  |
| 0 | transcript:Vitvi02g04302\_t001 |  |  |  |  |  |  |  |  |  |  |  |  |
| 0 | transcript:Vitvi02g04303\_t001 |  |  |  |  |  |  |  |  |  |  |  |  |
| 0 | transcript:Vitvi02g01004\_t001 |  |  |  |  |  |  |  |  |  |  |  |  |
| 0 | transcript:Vitvi02g01005\_t001 |  |  |  |  |  |  |  |  |  |  |  |  |
| 0 | transcript:Vitvi02g01011\_t001 |  |  |  |  |  |  |  |  |  |  |  |  |
| 0 | transcript:Vitvi02g01012\_t001 |  |  |  |  |  |  |  |  |  |  |  |  |
| 0 | transcript:Vitvi02g01584\_t001 |  |  |  |  |  |  |  |  |  |  |  |  |
| 0 | transcript:Vitvi02g04304\_t001 |  |  |  |  |  |  |  |  |  |  |  |  |
| 0 | transcript:Vitvi02g01015\_t001 |  |  |  |  |  |  |  |  |  |  |  |  |
| 0 | transcript:Vitvi02g01016\_t001 |  |  |  |  |  |  |  |  |  |  |  |  |
| 0 | transcript:Vitvi02g01307\_t001 |  |  |  |  |  |  |  |  |  |  |  |  |
| 0 | transcript:Vitvi02g04305\_t001 |  |  |  |  |  |  |  |  |  |  |  |  |
| 0 | transcript:Vitvi02g01019\_t001 |  |  |  |  |  |  |  |  |  |  |  |  |
| 0 | transcript:Vitvi02g01585\_t001 |  |  |  |  |  |  |  |  |  |  |  |  |
| 0 | transcript:Vitvi02g01308\_t001 |  |  |  |  |  |  |  |  |  |  |  |  |
| 0 | transcript:Vitvi02g01022\_t001 |  |  |  |  |  |  |  |  |  |  |  |  |
| 0 | transcript:Vitvi02g01024\_t001 |  |  |  |  |  |  |  |  |  |  |  |  |
| 0 | transcript:Vitvi02g01309\_t001 |  |  |  |  |  |  |  |  |  |  |  |  |
| 0 | transcript:Vitvi02g04306\_t001 |  |  |  |  |  |  |  |  |  |  |  |  |
| 0 | transcript:Vitvi02g04307\_t001 |  |  |  |  |  |  |  |  |  |  |  |  |
| 0 | transcript:Vitvi02g04308\_t001 |  |  |  |  |  |  |  |  |  |  |  |  |
| 0 | transcript:Vitvi02g04309\_t001 |  |  |  |  |  |  |  |  |  |  |  |  |
| 0 | transcript:Vitvi02g04310\_t001 |  |  |  |  |  |  |  |  |  |  |  |  |
| 0 | transcript:Vitvi02g04311\_t001 |  |  |  |  |  |  |  |  |  |  |  |  |
| 0 | transcript:Vitvi02g01594\_t001 |  |  |  |  |  |  |  |  |  |  |  |  |
| 0 | transcript:Vitvi02g04312\_t001 |  |  |  |  |  |  |  |  |  |  |  |  |
| 0 | transcript:Vitvi02g04313\_t001 |  |  |  |  |  |  |  |  |  |  |  |  |
| 0 | transcript:Vitvi02g04314\_t001 |  |  |  |  |  |  |  |  |  |  |  |  |
| 0 | transcript:Vitvi02g01038\_t001 |  |  |  |  |  |  |  |  |  |  |  |  |
| 0 | transcript:Vitvi02g01040\_t001 |  |  |  |  |  |  |  |  |  |  |  |  |
| 0 | transcript:Vitvi02g04315\_t001 |  |  |  |  |  |  |  |  |  |  |  |  |
| 0 | transcript:Vitvi02g04316\_t001 |  |  |  |  |  |  |  |  |  |  |  |  |
| 0 | transcript:Vitvi02g04317\_t001 |  |  |  |  |  |  |  |  |  |  |  |  |
| 0 | transcript:Vitvi02g04318\_t001 |  |  |  |  |  |  |  |  |  |  |  |  |
| 0 | transcript:Vitvi02g04319\_t001 |  |  |  |  |  |  |  |  |  |  |  |  |
| 0 | transcript:Vitvi02g01045\_t001 |  |  |  |  |  |  |  |  |  |  |  |  |
| 0 | transcript:Vitvi02g04320\_t001 |  |  |  |  |  |  |  |  |  |  |  |  |
| 0 | transcript:Vitvi02g01047\_t001 |  |  |  |  |  |  |  |  |  |  |  |  |
| 0 | transcript:Vitvi02g01050\_t001 |  |  |  |  |  |  |  |  |  |  |  |  |
| 0 | transcript:Vitvi02g04321\_t001 |  |  |  |  |  |  |  |  |  |  |  |  |
| 0 | transcript:Vitvi02g04322\_t001 |  |  |  |  |  |  |  |  |  |  |  |  |
| 0 | transcript:Vitvi02g01057\_t001 |  |  |  |  |  |  |  |  |  |  |  |  |
| 0 | transcript:Vitvi02g01602\_t001 |  |  |  |  |  |  |  |  |  |  |  |  |
| 0 | transcript:Vitvi02g04323\_t001 |  |  |  |  |  |  |  |  |  |  |  |  |
| 0 | transcript:Vitvi02g01605\_t001 |  |  |  |  |  |  |  |  |  |  |  |  |
| 0 | transcript:Vitvi02g01606\_t001 |  |  |  |  |  |  |  |  |  |  |  |  |
| 0 | transcript:Vitvi02g01607\_t001 |  |  |  |  |  |  |  |  |  |  |  |  |
| 0 | transcript:Vitvi02g04325\_t001 |  |  |  |  |  |  |  |  |  |  |  |  |
| 0 | transcript:Vitvi02g04326\_t001 |  |  |  |  |  |  |  |  |  |  |  |  |
| 0 | transcript:Vitvi02g01609\_t001 |  |  |  |  |  |  |  |  |  |  |  |  |
| 0 | transcript:Vitvi02g04327\_t001 |  |  |  |  |  |  |  |  |  |  |  |  |
| 0 | transcript:Vitvi02g01066\_t001 |  |  |  |  |  |  |  |  |  |  |  |  |
| 0 | transcript:Vitvi02g04328\_t001 |  |  |  |  |  |  |  |  |  |  |  |  |
| 0 | transcript:Vitvi02g04329\_t001 |  |  |  |  |  |  |  |  |  |  |  |  |
| 0 | transcript:Vitvi02g01067\_t001 |  |  |  |  |  |  |  |  |  |  |  |  |
| 0 | transcript:Vitvi02g04330\_t001 |  |  |  |  |  |  |  |  |  |  |  |  |
| 0 | transcript:Vitvi02g04331\_t001 |  |  |  |  |  |  |  |  |  |  |  |  |
| 0 | transcript:Vitvi02g04332\_t001 |  |  |  |  |  |  |  |  |  |  |  |  |
| 0 | transcript:Vitvi02g04333\_t001 |  |  |  |  |  |  |  |  |  |  |  |  |
| 0 | transcript:Vitvi02g04334\_t001 |  |  |  |  |  |  |  |  |  |  |  |  |
| 0 | transcript:Vitvi02g04335\_t001 |  |  |  |  |  |  |  |  |  |  |  |  |
| 0 | transcript:Vitvi02g01611\_t001 |  |  |  |  |  |  |  |  |  |  |  |  |
| 0 | transcript:Vitvi02g04336\_t001 |  |  |  |  |  |  |  |  |  |  |  |  |
| 0 | transcript:Vitvi02g04337\_t001 |  |  |  |  |  |  |  |  |  |  |  |  |
| 0 | transcript:Vitvi02g04338\_t001 |  |  |  |  |  |  |  |  |  |  |  |  |
| 0 | transcript:Vitvi02g04339\_t001 |  |  |  |  |  |  |  |  |  |  |  |  |
| 0 | transcript:Vitvi02g01613\_t001 |  |  |  |  |  |  |  |  |  |  |  |  |
| 0 | transcript:Vitvi02g01075\_t001 |  |  |  |  |  |  |  |  |  |  |  |  |
| 0 | transcript:Vitvi02g01614\_t001 |  |  |  |  |  |  |  |  |  |  |  |  |
| 0 | transcript:Vitvi02g01615\_t001 |  |  |  |  |  |  |  |  |  |  |  |  |
| 0 | transcript:Vitvi02g04340\_t001 |  |  |  |  |  |  |  |  |  |  |  |  |
| 0 | transcript:Vitvi02g01081\_t001 |  |  |  |  |  |  |  |  |  |  |  |  |
| 0 | transcript:Vitvi02g01082\_t001 |  |  |  |  |  |  |  |  |  |  |  |  |
| 0 | transcript:Vitvi02g01618\_t001 |  |  |  |  |  |  |  |  |  |  |  |  |
| 0 | transcript:Vitvi02g01621\_t001 |  |  |  |  |  |  |  |  |  |  |  |  |
| 0 | transcript:Vitvi02g01084\_t001 |  |  |  |  |  |  |  |  |  |  |  |  |
| 0 | transcript:Vitvi02g04341\_t001 |  |  |  |  |  |  |  |  |  |  |  |  |
| 0 | transcript:Vitvi02g04342\_t001 |  |  |  |  |  |  |  |  |  |  |  |  |
| 0 | transcript:Vitvi02g04343\_t001 |  |  |  |  |  |  |  |  |  |  |  |  |
| 0 | transcript:Vitvi02g01091\_t001 |  |  |  |  |  |  |  |  |  |  |  |  |
| 0 | transcript:Vitvi02g01092\_t001 |  |  |  |  |  |  |  |  |  |  |  |  |
| 0 | transcript:Vitvi02g01094\_t001 |  |  |  |  |  |  |  |  |  |  |  |  |
| 0 | transcript:Vitvi02g04344\_t001 |  |  |  |  |  |  |  |  |  |  |  |  |
| 0 | transcript:Vitvi02g01098\_t001 |  |  |  |  |  |  |  |  |  |  |  |  |
| 0 | transcript:Vitvi02g01100\_t001 |  |  |  |  |  |  |  |  |  |  |  |  |
| 0 | transcript:Vitvi02g01101\_t001 |  |  |  |  |  |  |  |  |  |  |  |  |
| 0 | transcript:Vitvi02g04345\_t001 |  |  |  |  |  |  |  |  |  |  |  |  |
| 0 | transcript:Vitvi02g04346\_t001 |  |  |  |  |  |  |  |  |  |  |  |  |
| 0 | transcript:Vitvi02g04347\_t001 |  |  |  |  |  |  |  |  |  |  |  |  |
| 0 | transcript:Vitvi02g04348\_t001 |  |  |  |  |  |  |  |  |  |  |  |  |
| 0 | transcript:Vitvi02g01113\_t001 |  |  |  |  |  |  |  |  |  |  |  |  |
| 0 | transcript:Vitvi02g04349\_t001 |  |  |  |  |  |  |  |  |  |  |  |  |
| 0 | transcript:Vitvi02g01118\_t001 |  |  |  |  |  |  |  |  |  |  |  |  |
| 0 | transcript:Vitvi02g01121\_t001 |  |  |  |  |  |  |  |  |  |  |  |  |
| 0 | transcript:Vitvi02g04350\_t001 |  |  |  |  |  |  |  |  |  |  |  |  |
| 0 | transcript:Vitvi02g04351\_t001 |  |  |  |  |  |  |  |  |  |  |  |  |
| 0 | transcript:Vitvi02g04352\_t001 |  |  |  |  |  |  |  |  |  |  |  |  |
| 0 | transcript:Vitvi02g01126\_t001 |  |  |  |  |  |  |  |  |  |  |  |  |
| 0 | transcript:Vitvi02g04353\_t001 |  |  |  |  |  |  |  |  |  |  |  |  |
| 0 | transcript:Vitvi02g04354\_t001 |  |  |  |  |  |  |  |  |  |  |  |  |
| 0 | transcript:Vitvi02g04355\_t001 |  |  |  |  |  |  |  |  |  |  |  |  |
| 0 | transcript:Vitvi02g01133\_t001 |  |  |  |  |  |  |  |  |  |  |  |  |
| 0 | transcript:Vitvi02g04356\_t001 |  |  |  |  |  |  |  |  |  |  |  |  |
| 0 | transcript:Vitvi02g04357\_t001 |  |  |  |  |  |  |  |  |  |  |  |  |
| 0 | transcript:Vitvi02g01138\_t001 |  |  |  |  |  |  |  |  |  |  |  |  |
| 0 | transcript:Vitvi02g04358\_t001 |  |  |  |  |  |  |  |  |  |  |  |  |
| 0 | transcript:Vitvi02g01139\_t001 |  |  |  |  |  |  |  |  |  |  |  |  |
| 0 | transcript:Vitvi02g04359\_t001 |  |  |  |  |  |  |  |  |  |  |  |  |
| 0 | transcript:Vitvi02g01140\_t001 |  |  |  |  |  |  |  |  |  |  |  |  |
| 0 | transcript:Vitvi02g04360\_t001 |  |  |  |  |  |  |  |  |  |  |  |  |
| 0 | transcript:Vitvi02g04361\_t001 |  |  |  |  |  |  |  |  |  |  |  |  |
| 0 | transcript:Vitvi02g01143\_t002 |  |  |  |  |  |  |  |  |  |  |  |  |
| 0 | transcript:Vitvi02g04362\_t001 |  |  |  |  |  |  |  |  |  |  |  |  |
| 0 | transcript:Vitvi02g04363\_t001 |  |  |  |  |  |  |  |  |  |  |  |  |
| 0 | transcript:Vitvi02g01149\_t001 |  |  |  |  |  |  |  |  |  |  |  |  |
| 0 | transcript:Vitvi02g01150\_t002 |  |  |  |  |  |  |  |  |  |  |  |  |
| 0 | transcript:Vitvi02g01152\_t001 |  |  |  |  |  |  |  |  |  |  |  |  |
| 0 | transcript:Vitvi02g01153\_t001 |  |  |  |  |  |  |  |  |  |  |  |  |
| 0 | transcript:Vitvi02g01154\_t001 |  |  |  |  |  |  |  |  |  |  |  |  |
| 0 | transcript:Vitvi02g04364\_t001 |  |  |  |  |  |  |  |  |  |  |  |  |
| 0 | transcript:Vitvi02g04365\_t001 |  |  |  |  |  |  |  |  |  |  |  |  |
| 0 | transcript:Vitvi02g01158\_t001 |  |  |  |  |  |  |  |  |  |  |  |  |
| 0 | transcript:Vitvi02g01160\_t001 |  |  |  |  |  |  |  |  |  |  |  |  |
| 0 | transcript:Vitvi02g04366\_t001 |  |  |  |  |  |  |  |  |  |  |  |  |
| 0 | transcript:Vitvi02g01161\_t001 |  |  |  |  |  |  |  |  |  |  |  |  |
| 0 | transcript:Vitvi02g01162\_t001 |  |  |  |  |  |  |  |  |  |  |  |  |
| 0 | transcript:Vitvi02g04367\_t001 |  |  |  |  |  |  |  |  |  |  |  |  |
| 0 | transcript:Vitvi02g01163\_t001 |  |  |  |  |  |  |  |  |  |  |  |  |
| 0 | transcript:Vitvi02g01164\_t001 |  |  |  |  |  |  |  |  |  |  |  |  |
| 0 | transcript:Vitvi02g01166\_t001 |  |  |  |  |  |  |  |  |  |  |  |  |
| 0 | transcript:Vitvi02g01167\_t001 |  |  |  |  |  |  |  |  |  |  |  |  |
| 0 | transcript:Vitvi02g04368\_t001 |  |  |  |  |  |  |  |  |  |  |  |  |
| 0 | transcript:Vitvi02g04369\_t001 |  |  |  |  |  |  |  |  |  |  |  |  |
| 0 | transcript:Vitvi02g01171\_t001 |  |  |  |  |  |  |  |  |  |  |  |  |
| 0 | transcript:Vitvi02g01172\_t001 |  |  |  |  |  |  |  |  |  |  |  |  |
| 0 | transcript:Vitvi02g01173\_t001 |  |  |  |  |  |  |  |  |  |  |  |  |
| 0 | transcript:Vitvi02g04370\_t001 |  |  |  |  |  |  |  |  |  |  |  |  |
| 0 | transcript:Vitvi02g04371\_t001 |  |  |  |  |  |  |  |  |  |  |  |  |
| 0 | transcript:Vitvi02g01177\_t001 |  |  |  |  |  |  |  |  |  |  |  |  |
| 0 | transcript:Vitvi02g01179\_t001 |  |  |  |  |  |  |  |  |  |  |  |  |
| 0 | transcript:Vitvi02g01180\_t001 |  |  |  |  |  |  |  |  |  |  |  |  |
| 0 | transcript:Vitvi02g01182\_t001 |  |  |  |  |  |  |  |  |  |  |  |  |
| 0 | transcript:Vitvi02g04372\_t001 |  |  |  |  |  |  |  |  |  |  |  |  |
| 0 | transcript:Vitvi02g01640\_t001 |  |  |  |  |  |  |  |  |  |  |  |  |
| 0 | transcript:Vitvi02g01183\_t001 |  |  |  |  |  |  |  |  |  |  |  |  |
| 0 | transcript:Vitvi02g04373\_t001 |  |  |  |  |  |  |  |  |  |  |  |  |
| 0 | transcript:Vitvi02g04374\_t001 |  |  |  |  |  |  |  |  |  |  |  |  |
| 0 | transcript:Vitvi02g04375\_t001 |  |  |  |  |  |  |  |  |  |  |  |  |
| 0 | transcript:Vitvi02g04376\_t001 |  |  |  |  |  |  |  |  |  |  |  |  |
| 0 | transcript:Vitvi02g04377\_t001 |  |  |  |  |  |  |  |  |  |  |  |  |
| 0 | transcript:Vitvi02g01642\_t001 |  |  |  |  |  |  |  |  |  |  |  |  |
| 0 | transcript:Vitvi02g04378\_t001 |  |  |  |  |  |  |  |  |  |  |  |  |
| 0 | transcript:Vitvi02g04379\_t001 |  |  |  |  |  |  |  |  |  |  |  |  |
| 0 | transcript:Vitvi02g01187\_t001 |  |  |  |  |  |  |  |  |  |  |  |  |
| 0 | transcript:Vitvi02g04380\_t001 |  |  |  |  |  |  |  |  |  |  |  |  |
| 0 | transcript:Vitvi02g01189\_t001 |  |  |  |  |  |  |  |  |  |  |  |  |
| 0 | transcript:Vitvi02g01190\_t001 |  |  |  |  |  |  |  |  |  |  |  |  |
| 0 | transcript:Vitvi02g04381\_t001 |  |  |  |  |  |  |  |  |  |  |  |  |
| 0 | transcript:Vitvi02g01191\_t001 |  |  |  |  |  |  |  |  |  |  |  |  |
| 0 | transcript:Vitvi02g01192\_t001 |  |  |  |  |  |  |  |  |  |  |  |  |
| 0 | transcript:Vitvi02g01648\_t001 |  |  |  |  |  |  |  |  |  |  |  |  |
| 0 | transcript:Vitvi02g01193\_t001 |  |  |  |  |  |  |  |  |  |  |  |  |
| 0 | transcript:Vitvi02g01201\_t001 |  |  |  |  |  |  |  |  |  |  |  |  |
| 0 | transcript:Vitvi02g01203\_t001 |  |  |  |  |  |  |  |  |  |  |  |  |
| 0 | transcript:Vitvi02g01650\_t001 |  |  |  |  |  |  |  |  |  |  |  |  |
| 0 | transcript:Vitvi02g01204\_t001 |  |  |  |  |  |  |  |  |  |  |  |  |
| 0 | transcript:Vitvi02g01205\_t001 |  |  |  |  |  |  |  |  |  |  |  |  |
| 0 | transcript:Vitvi02g04382\_t001 |  |  |  |  |  |  |  |  |  |  |  |  |
| 0 | transcript:Vitvi02g01207\_t001 |  |  |  |  |  |  |  |  |  |  |  |  |
| 0 | transcript:Vitvi02g01208\_t001 |  |  |  |  |  |  |  |  |  |  |  |  |
| 0 | transcript:Vitvi02g01209\_t001 |  |  |  |  |  |  |  |  |  |  |  |  |
| 0 | transcript:Vitvi02g01651\_t001 |  |  |  |  |  |  |  |  |  |  |  |  |
| 0 | transcript:Vitvi02g01652\_t001 |  |  |  |  |  |  |  |  |  |  |  |  |
| 0 | transcript:Vitvi02g01210\_t001 |  |  |  |  |  |  |  |  |  |  |  |  |
| 0 | transcript:Vitvi02g01653\_t001 |  |  |  |  |  |  |  |  |  |  |  |  |
| 0 | transcript:Vitvi02g01211\_t001 |  |  |  |  |  |  |  |  |  |  |  |  |
| 0 | transcript:Vitvi02g04383\_t001 |  |  |  |  |  |  |  |  |  |  |  |  |
| 0 | transcript:Vitvi02g01214\_t001 |  |  |  |  |  |  |  |  |  |  |  |  |
| 0 | transcript:Vitvi02g01215\_t001 |  |  |  |  |  |  |  |  |  |  |  |  |
| 0 | transcript:Vitvi02g04384\_t001 |  |  |  |  |  |  |  |  |  |  |  |  |
| 0 | transcript:Vitvi02g01220\_t001 |  |  |  |  |  |  |  |  |  |  |  |  |
| 0 | transcript:Vitvi02g01222\_t001 |  |  |  |  |  |  |  |  |  |  |  |  |
| 0 | transcript:Vitvi02g01224\_t001 |  |  |  |  |  |  |  |  |  |  |  |  |
| 0 | transcript:Vitvi02g04385\_t001 |  |  |  |  |  |  |  |  |  |  |  |  |
| 0 | transcript:Vitvi02g01225\_t001 |  |  |  |  |  |  |  |  |  |  |  |  |
| 0 | transcript:Vitvi02g01227\_t001 |  |  |  |  |  |  |  |  |  |  |  |  |
| 0 | transcript:Vitvi02g01661\_t001 |  |  |  |  |  |  |  |  |  |  |  |  |
| 0 | transcript:Vitvi02g01228\_t001 |  |  |  |  |  |  |  |  |  |  |  |  |
| 0 | transcript:Vitvi02g01230\_t001 |  |  |  |  |  |  |  |  |  |  |  |  |
| 0 | transcript:Vitvi02g01662\_t001 |  |  |  |  |  |  |  |  |  |  |  |  |
| 0 | transcript:Vitvi02g01231\_t002 |  |  |  |  |  |  |  |  |  |  |  |  |
| 0 | transcript:Vitvi02g01232\_t001 |  |  |  |  |  |  |  |  |  |  |  |  |
| 0 | transcript:Vitvi02g01233\_t001 |  |  |  |  |  |  |  |  |  |  |  |  |
| 0 | transcript:Vitvi02g01235\_t001 |  |  |  |  |  |  |  |  |  |  |  |  |
| 0 | transcript:Vitvi02g01236\_t005 |  |  |  |  |  |  |  |  |  |  |  |  |
| 0 | transcript:Vitvi02g04386\_t001 |  |  |  |  |  |  |  |  |  |  |  |  |
| 0 | transcript:Vitvi02g04387\_t001 |  |  |  |  |  |  |  |  |  |  |  |  |
| 0 | transcript:Vitvi02g04388\_t001 |  |  |  |  |  |  |  |  |  |  |  |  |
| 0 | transcript:Vitvi02g01237\_t001 |  |  |  |  |  |  |  |  |  |  |  |  |
| 0 | transcript:Vitvi02g04389\_t001 |  |  |  |  |  |  |  |  |  |  |  |  |
| 0 | transcript:Vitvi02g04390\_t001 |  |  |  |  |  |  |  |  |  |  |  |  |
| 0 | transcript:Vitvi02g01240\_t001 |  |  |  |  |  |  |  |  |  |  |  |  |
| 0 | transcript:Vitvi02g01241\_t001 |  |  |  |  |  |  |  |  |  |  |  |  |
| 0 | transcript:Vitvi02g04391\_t001 |  |  |  |  |  |  |  |  |  |  |  |  |
| 0 | transcript:Vitvi02g04392\_t001 |  |  |  |  |  |  |  |  |  |  |  |  |
| 0 | transcript:Vitvi02g01243\_t001 |  |  |  |  |  |  |  |  |  |  |  |  |
| 0 | transcript:Vitvi02g04393\_t001 |  |  |  |  |  |  |  |  |  |  |  |  |
| 0 | transcript:Vitvi02g04394\_t001 |  |  |  |  |  |  |  |  |  |  |  |  |
| 0 | transcript:Vitvi02g04395\_t001 |  |  |  |  |  |  |  |  |  |  |  |  |
| 0 | transcript:Vitvi02g04396\_t001 |  |  |  |  |  |  |  |  |  |  |  |  |
| 0 | transcript:Vitvi02g04397\_t001 |  |  |  |  |  |  |  |  |  |  |  |  |
| 0 | transcript:Vitvi02g01245\_t001 |  |  |  |  |  |  |  |  |  |  |  |  |
| 0 | transcript:Vitvi02g01246\_t001 |  |  |  |  |  |  |  |  |  |  |  |  |
| 0 | transcript:Vitvi02g04398\_t001 |  |  |  |  |  |  |  |  |  |  |  |  |
| 0 | transcript:Vitvi02g04399\_t001 |  |  |  |  |  |  |  |  |  |  |  |  |
| 0 | transcript:Vitvi02g01248\_t001 |  |  |  |  |  |  |  |  |  |  |  |  |
| 0 | transcript:Vitvi02g04400\_t001 |  |  |  |  |  |  |  |  |  |  |  |  |
| 0 | transcript:Vitvi02g01251\_t001 |  |  |  |  |  |  |  |  |  |  |  |  |
| 0 | transcript:Vitvi02g01664\_t001 |  |  |  |  |  |  |  |  |  |  |  |  |
| 0 | transcript:Vitvi02g01252\_t001 |  |  |  |  |  |  |  |  |  |  |  |  |
| 0 | transcript:Vitvi02g04401\_t001 |  |  |  |  |  |  |  |  |  |  |  |  |
| 0 | transcript:Vitvi02g01253\_t001 |  |  |  |  |  |  |  |  |  |  |  |  |
| 0 | transcript:Vitvi02g04402\_t001 |  |  |  |  |  |  |  |  |  |  |  |  |
| 0 | transcript:Vitvi02g04403\_t001 |  |  |  |  |  |  |  |  |  |  |  |  |
| 0 | transcript:Vitvi02g04404\_t001 |  |  |  |  |  |  |  |  |  |  |  |  |
| 0 | transcript:Vitvi02g01261\_t001 |  |  |  |  |  |  |  |  |  |  |  |  |
| 0 | transcript:Vitvi02g01665\_t001 |  |  |  |  |  |  |  |  |  |  |  |  |
| 0 | transcript:Vitvi02g04405\_t001 |  |  |  |  |  |  |  |  |  |  |  |  |
| 0 | transcript:Vitvi02g01264\_t001 |  |  |  |  |  |  |  |  |  |  |  |  |
| 0 | transcript:Vitvi02g01266\_t001 |  |  |  |  |  |  |  |  |  |  |  |  |
| 0 | transcript:Vitvi02g01267\_t001 |  |  |  |  |  |  |  |  |  |  |  |  |
| 0 | transcript:Vitvi02g01268\_t001 |  |  |  |  |  |  |  |  |  |  |  |  |
| 0 | transcript:Vitvi02g01666\_t001 |  |  |  |  |  |  |  |  |  |  |  |  |
| 0 | transcript:Vitvi02g01269\_t001 |  |  |  |  |  |  |  |  |  |  |  |  |
| 0 | transcript:Vitvi02g01270\_t001 |  |  |  |  |  |  |  |  |  |  |  |  |
| 0 | transcript:Vitvi02g04406\_t001 |  |  |  |  |  |  |  |  |  |  |  |  |
| 0 | transcript:Vitvi02g01668\_t001 |  |  |  |  |  |  |  |  |  |  |  |  |
| 0 | transcript:Vitvi02g01272\_t001 |  |  |  |  |  |  |  |  |  |  |  |  |
| 0 | transcript:Vitvi02g01273\_t001 |  |  |  |  |  |  |  |  |  |  |  |  |
| 0 | transcript:Vitvi02g01274\_t001 |  |  |  |  |  |  |  |  |  |  |  |  |
| 0 | transcript:Vitvi02g01669\_t001 |  |  |  |  |  |  |  |  |  |  |  |  |
| 0 | transcript:Vitvi02g01670\_t001 |  |  |  |  |  |  |  |  |  |  |  |  |
| 0 | transcript:Vitvi02g01275\_t001 |  |  |  |  |  |  |  |  |  |  |  |  |
| 0 | transcript:Vitvi02g01276\_t001 |  |  |  |  |  |  |  |  |  |  |  |  |
| 0 | transcript:Vitvi02g04407\_t001 |  |  |  |  |  |  |  |  |  |  |  |  |
| 0 | transcript:Vitvi02g01277\_t001 |  |  |  |  |  |  |  |  |  |  |  |  |
| 0 | transcript:Vitvi02g01278\_t001 |  |  |  |  |  |  |  |  |  |  |  |  |
| 0 | transcript:Vitvi02g01279\_t001 |  |  |  |  |  |  |  |  |  |  |  |  |
| 0 | transcript:Vitvi02g01280\_t001 |  |  |  |  |  |  |  |  |  |  |  |  |
| 0 | transcript:Vitvi02g04408\_t001 |  |  |  |  |  |  |  |  |  |  |  |  |
| 0 | transcript:Vitvi02g01283\_t001 |  |  |  |  |  |  |  |  |  |  |  |  |
| 0 | transcript:Vitvi02g01284\_t001 |  |  |  |  |  |  |  |  |  |  |  |  |
| 0 | transcript:Vitvi02g01286\_t001 |  |  |  |  |  |  |  |  |  |  |  |  |
| 0 | transcript:Vitvi02g01288\_t001 |  |  |  |  |  |  |  |  |  |  |  |  |
| 0 | transcript:Vitvi02g04409\_t001 |  |  |  |  |  |  |  |  |  |  |  |  |
| 0 | transcript:Vitvi02g01290\_t002 |  |  |  |  |  |  |  |  |  |  |  |  |
